# Competitive Effects of Anions on Protein Solvation by Aqueous Ionic Liquids

**DOI:** 10.1021/acs.jpcb.4c03735

**Published:** 2024-08-02

**Authors:** Vinicius Piccoli, Leandro Martínez

**Affiliations:** Institute of Chemistry and Center for Computing in Engineering & Science, Universidade Estadual de Campinas (UNICAMP), Campinas 13083-872, SP, Brazil

## Abstract

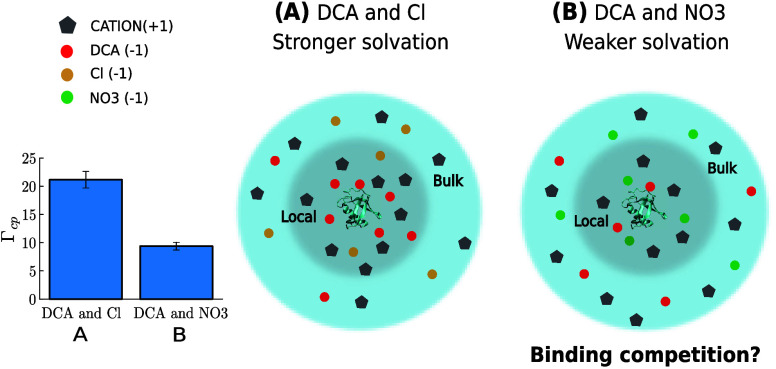

The present study utilizes molecular dynamics simulations to examine how different anions compete for protein solvation in aqueous solutions of ionic liquids (ILs). Ubiquitin is used as model protein and studied in IL mixtures sharing the same cation, 1-ethyl-3-methylimidazolium (EMIM), and two different anions in the same solution, from combinations of dicyanamide (DCA), chloride (Cl), nitrate (NO_3_), and tetrafluoroborate (BF_4_). Our findings reveal that specific interactions between anions and the protein are paramount in IL solvation, but that combinations of anions are not additive. For example, DCA exhibits a remarkable ability to form hydrogen bonds with the protein, resulting in a significantly stronger preferential binding to the protein than other anions. However, the combination of DCA with NO_3_, which also forms hydrogen bonds with the protein, results in a smaller preferential solvation of the protein than the combination of DCA with chloride ions, which are weaker binders. Thus, combining anions with varying affinities for the protein surface modulates the overall ion accumulation through nonadditive mechanisms, highlighting the importance of the understanding of competition for specific interaction sites, cooperative binding, bulk-solution affinity, and overall charge compensations, on the overall solvation capacity of the solution. Such knowledge may allow for the design of novel IL-based processes in biotechnology and material science, where fine-tuning protein solvation is crucial for optimizing performance and functionality.

## Introduction

Understanding how ions and proteins interact in solution is essential to figuring out critical molecular processes that broadly affect biological and biophysical fields. The influence of ions on protein stability has been the focus of extensive research efforts since the pioneering studies of Hofmeister and colleagues in 1888.^1−4^ However, despite decades of investigation, the precise nature of ion-protein interactions remains a subject of ongoing debate, particularly for complex ions and electrolyte mixtures.^5,6^ This complexity arises from the intricate interplay of interactions between ions, water, and the protein surface.

For instance, the knowledge of ion charges or concentrations is insufficient to understand ion-protein interactions; the identity of ions also plays a pivotal role.^7−12^ This phenomenon, known as the specific ion effect, has been systematically explored by researchers such as Lewith and Hofmeister. They observed that different ions exhibit varying degrees of effectiveness in precipitating proteins from aqueous solutions of blood serum and hen egg white.^1,2^ This ordering of ions has been observed in biological,^7,13^ polymer,^14−17^ and nonaqueous^9,18,19^ systems and is known as the Hofmeister series. Its importance underscores the significance of ion identity in shaping solute (especially proteins) behavior.^20^

These pioneering studies shed light on the impact of simple salts on protein and polymer structures,^21,22^ but the mixture of two or more salts in an aqueous solution, each containing ions with distinct chemical nature, can give rise to nonadditive combined effects.^23^ Exploring complex ions, such as those found in ionic liquids, becomes paramount in this context. Ionic liquids, composed of large and asymmetric ions, offer a diverse array of intermolecular interactions with water and the various chemical moieties commonly found on protein surfaces.^24^ These versatile liquids have gradually replaced conventional and hazardous organic solvents in applications related to protein stability and tuning enzyme activity, for example.^23,25−28^ However, fewer studies have delved into systems involving salt mixtures compared to single-salt solutions.^23^

Ionic liquids have diverse applications owing to their structural, compositional, and physicochemical diversity, making them ideal candidates for exploring the intricate effects of multielectrolyte interactions on macromolecules. Furthermore, understanding how ionic liquids interact with other solutes can inform tailored applications. In this study, we employ molecular dynamics simulations to investigate the competition of anions of a distinct chemical nature for the interactions with the Ubiquitin surface. We analyze the solvation structure using minimum-distance distribution functions (MDDFs) and the Kirkwood-Buff theory of solvation.

## Methods

Equilibrium molecular dynamics simulations were performed using GROMACS 2023.3 software,^29,30^ and the initial configurations of the systems were generated using Packmol.^31,32^ The ionic liquid (IL) parameters were described using the virtual-site optimized potentials for liquid simulations (OPLS) force fields,^33^ while the protein was modeled with the OPLS-AA force fields.^34^ The TIP3P model was utilized to represent water molecules.^35^ The use of TIP3P in ionic liquid (IL) mixtures has already been documented in the literature.^36,37^ Numerical integration of the equations of motion was executed using the Verlet leapfrog algorithm with a time step of 2 fs.

A cutoff of 1.0 nm was employed for short-range electrostatic and Lennard–Jones interactions. We computed Long-range electrostatic interactions using the particle-mesh Ewald method,^38^ featuring a fourth-order interpolation and a grid spacing of 0.16 nm. The simulation temperature was maintained at 300 K, with temperature control implemented using the modified Berendsen thermostat and a relaxation time of 0.1 ps.^39,40^ The Parrinello–Rahman algorithm was utilized to keep the pressure constant at 1 bar, with a relaxation time of 2 ps and an isothermal compressibility of 4.5 × 10^–5^ bar.^41,42^

We initially subjected each system to an energy minimization stage comprising 50,000 Steepest-Descent steps, holding all protein coordinates fixed. This was followed by thermal equilibration in the NVT ensemble for 1 ns and then 5 ns of molecular dynamics under isothermic-isobaric (NPT) conditions, with the protein backbone constrained by applying soft harmonic constraints with a 10 kJ mol^–1^ Å^–2^ force constant to the Cα atoms of the protein structures. Next, we lifted the structural restrictions and conducted 1 ns simulations under constant pressure and temperature, from which production simulations followed for 10 ns, in the NPT ensemble. To ensure adequate sampling, we independently performed 20 simulations following this protocol for the system, following the methodology of previous studies for the proper sampling of solvent conformations around the folded state of a protein.^43−45^ Within the time scale of the simulations performed, protein structural variations were minimal, thus ensuring the focus remained on solvent organization surrounding the intact folded state of the protein. As depicted in Figure S8 of the Supporting Information, these structural perturbations were constrained to less than approximately 1.5 Å, underscoring the stability of the protein conformation within the simulated time frame.

The ComplexMixtures.jl^46^ package was used to compute minimum-distance distribution functions (MDDFs),^47^ Kirkwood–Buff (KB) integrals, and associated preferential interaction parameters.^43−45^ The density of solvent molecules in each distance was derived from the average number of minimum-distances at each 0.1 Å bin. MDDFs are advantageous in representing interactions among molecules of irregular shapes, because the nature of the minimum-distances takes automatically into consideration the complexity of the structures.^43−45^ The advantage of MDDFs stems from their focus on minimum distances, enabling a clearer view of local interactions compared to radial distribution functions (RDFs). RDFs measure distances between centers of mass or specific atoms, which can obscure interactions at closer distances because the centers are often significantly removed from the molecule’s surface, especially in larger molecules. This distinction allows MDDFs to provide a more accurate representation of interactions, especially in environments where molecular proximity plays a crucial role (in the case of Chloride, which is monatomic, the MDDFs reduce to “proximal distribution functions”, which take into account the distance of a single reference position in the solvent molecule^48^). Additionally, the atoms, or atom types, that satisfy the minimum distances at each instant can be annotated, and then the total MDDF can be decomposed in the fraction of the total density that results from the contribution of each type of atom.

The Kirkwood–Buff integrals were calculated up to a finite distance *R* with eq 1(47)
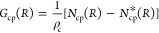
1where ρ_c_ is the bulk concentration of the solvent species in the solution, *N*_cp_(*R*) is the number of solute–solvent minimum-distances smaller than *R* in the simulation and *N*_cp_^*^(*R*) is the minimum-distance count in a reference state without solute–solvent interactions but with the same density of the bulk solvent.^46,47^*G*_cp_(*R*) converges when *R* is large enough such that the presence of the solute does not affect the distribution of the solvent molecules.

Adequate KB integral convergence was observed with *R* = 20 Å in all systems (which is unusually large,^47,49^ demands large solvation boxes, and was required because of the size and electrostatic nature of the IL ions). The solution volume closer to the solute than this distance was therefore considered the “protein domain”, i.e. the region of the solution where the solution structure is influenced by the presence of the protein. The volume outside this domain contains the mixture of cosolvents and is used to deduce the structure and thermodynamic properties of the solution without the protein. The effective bulk concentrations of the solutions was obtained from the simulations, by computing the density of each solvent in the region between 20 and 30 Å from the protein surface, thus from an open subvolume of the system, providing a finite-size correction to the computation of KB integrals.^50^

To compute the bulk solution hydration numbers of ions (Supporting Information Figure S9 and Tables S5 and S6), we implemented the *bulk_coordination* function in the MolSimToolkit.jl (http://m3g.github.io/MolSimToolkit.jl) package. This function uses a fast cell list implementation^51^ to compute all the water molecules within a radius of each ion, as a function of the distance to the protein. We computed hydration numbers of dicyanamide (DCA) within 5 and 10 Å, to obtain insights into the effect of the bulk solution composition into the water affinity to the ions.

**Figure 1:**
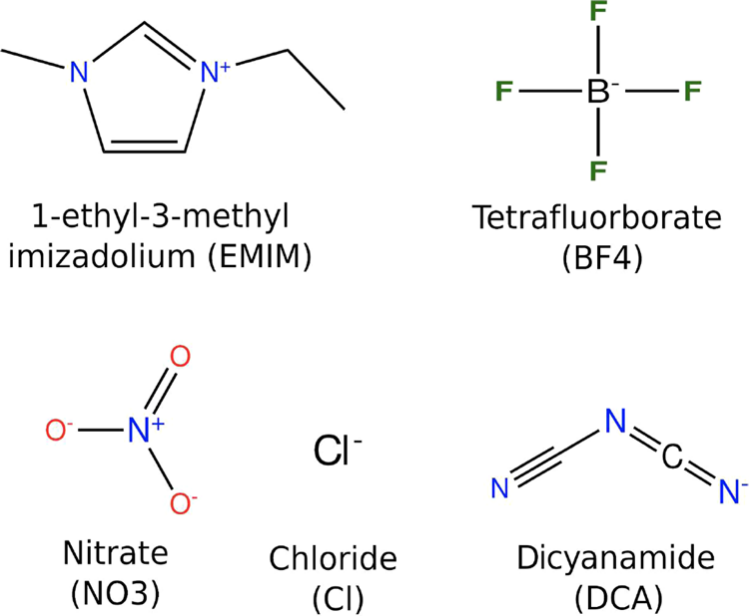
Molecular structures of the ions studied as components of the aqueous ionic liquids.

In this work, we investigated the solvation of Ubiquitin (PDB id. 1UBQ(52)) in ionic liquids (ILs) composed of combinations of the cation EMIM (1-ethyl-3-methylimidazolium, EMIM^+^) with of four different anions: DCA (dicyanamide, DCA^–^), BF_4_ (tetrafluoroborate, BF_4_^–^), NO_3_ (nitrate, NO_3_^–^), and Cl (chloride, Cl^–^). Figure 1 provides the molecular structures of all ions used in this work. The notation will omit charges and subscripts to simplify references to the ion throughout the paper. Two different compositions were simulated: systems containing a single ionic liquid and systems with a mixture of two ionic liquids sharing EMIM as the common cation. Since Ubiquitin does not possess a net charge, adding counterions for neutralization was unnecessary. In solutions composed solely of IL ions, charge neutrality allows us to treat these ions as equivalent species when computing Kirkwood–Buff (KB) integrals. Consequently, the system can be considered a pseudothree-component mixture. This simplifies the application of KB theory, especially compared to systems with non-neutral solutes or multiple ionic species.^53−55^

Simulations were conducted at total ionic liquid reference concentrations of 0.5, 1.0, 1.5, 2.0, 2.5, and 3.0 mol L^–1^. Each system’s concentration was recalculated from the NPT production using the bulk region of the simulation box. Table 1 presents the box sides, number of ions, and postequilibration concentrations for the systems containing EMIMDCA + EMIMCl solutions. Corresponding data for other IL solutions are available in Supporting Information Tables S1 and S2. From the bulk concentrations of the anions, it is possible to anticipate the preferential interactions with the protein, which will be discussed in the next section.

**Table 1:** Simulation Boxes Used and Concentrations for Water and Ions after the NPT Equilibration for EMIMDCA + EMIMCl Systems^a^

		number of components		bulk concentrations (mol L^–1^)			
ionic liquid mixture	box sides (Å)	ions	water	IL target	water	EMIM	DCA/Cl
EMIMDCA + EMIMCl	95.0	252	25,659	0.5	51.16 ± 0.02	0.48 ± 0.02	0.22 ± 0.01:0.25 ± 0.02
	95.0	502	23,410	1.0	46.51 ± 0.03	0.97 ± 0.03	0.46 ± 0.02:0.52 ± 0.01
	95.0	752	21,161	1.5	42.01 ± 0.05	1.49 ± 0.02	0.72 ± 0.01:0.78 ± 0.02
	95.0	1004	18,894	2.0	38.26 ± 0.09	2.05 ± 0.03	0.98 ± 0.02:1.06 ± 0.01
	95.0	1254	16,646	2.5	34.5 ± 0.1	2.62 ± 0.01	1.29 ± 0.01:1.33 ± 0.01
	95.0	1504	14,397	3.0	30.29 ± 0.03	3.19 ± 0.03	1.55 ± 0.01:1.63 ± 0.01

a Fluctuations were calculated using the standard error of the mean calculated for each concentration’s 20 simulations. The corresponding data for the other IL solutions are shown in Supporting Information Tables S1 and S2. Each system contains an equal number of the cations (EMIM) and total anions (DCA + Cl), and the table reports the total number of ions in the solution. DCA and Cl ions are present in equal amounts in each system. The effective bulk concentrations are displayed for each system component

## Results and Discussion

### Solvation of the Systems with Multiple Anions

In this section, we focus on the interactions observed in systems containing 2.0 mol L^–1^ solutions of ionic liquid (IL) mixtures with various anions alongside the EMIM cation. Most conclusions obtained for these systems can be extrapolated to other concentrations, with exceptions mentioned when appropriate. The data for other systems and concentrations is available as Supporting Information. We examine solutions containing single IL (a single anion) as reference states relative to solutions with anion mixtures. This section focuses on the MDDFs and KB integrals, which are instrumental in computing the preferential interaction parameters discussed in later sections.

**Figure 2:**
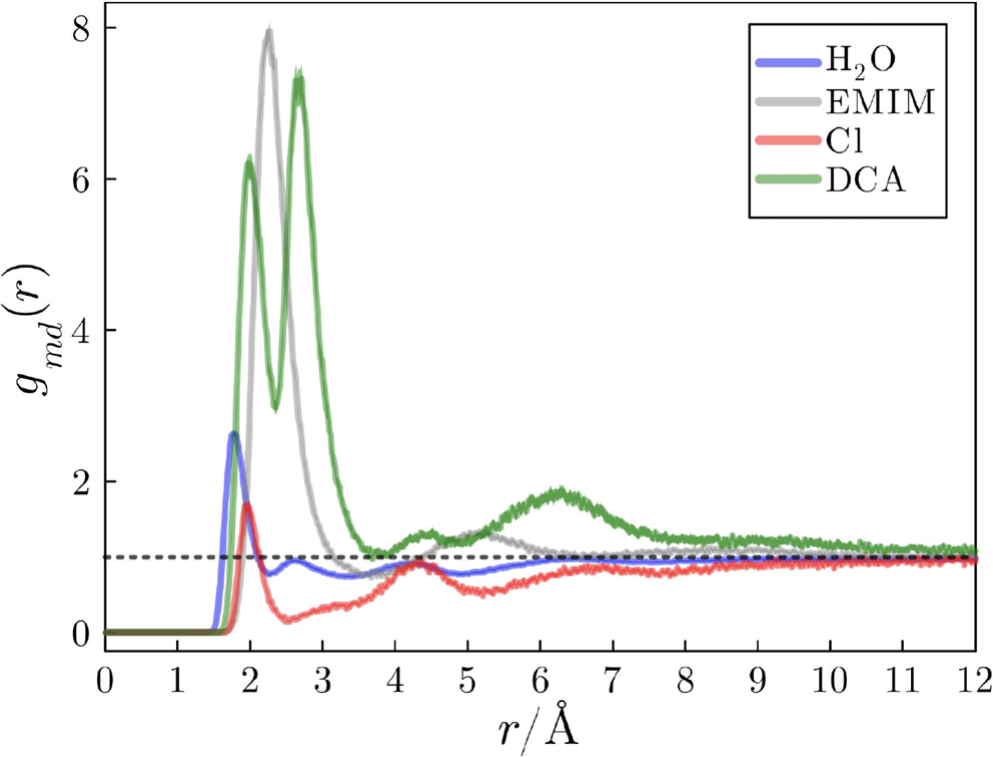
Ubiquitin-ion minimum-distance distribution functions (MDDFs) for DCA, Cl, EMIM, and water in the EMIMDCA + EMIMCl aqueous solution. Hydrogen-bonding interactions are associated with peaks of the distributions at ∼1.9 Å, and are present for DCA, and water. Strong electrostatic interactions appear at ∼2.0 Å and form the most relevant Cl peak, but are also present for the other ions. Less specific interactions are responsible for peaks at greater distances.

The MDDFs provide a molecular picture of the interactions between the protein and the ionic liquids. For example, the MDDFs in Figure 2 portray the interactions with the protein of each component of the system with 2.0 mol L^–1^ of EMIMDCA + EMIMCl. DCA is an anion that exhibits a pronounced affinity for the protein’s surface, as found in previous studies.^43−45^ DCA distribution, represented by the green curve in Figure 2, reveals two significant accumulation peaks within a distance of up to 4 Å. The initial peak, at approximately 1.9 Å, arises from hydrogen bonds formed via the nitrogen atoms of DCA. In contrast, the second peak encompasses a range of interactions, including direct dispersive-like interactions between DCA and the protein and interactions mediated by other ions or molecules positioned between DCA and the protein surface atoms. The Lennard–Jones and electrostatic interaction energies between the ions and the protein, computed within a 10 Å radius from the protein surface, are detailed in Table S4 of the Supporting Information. The broad peak extending from 5 to 7.5 Å indicates that the correlation between DCA distribution and the presence of the protein persists up to large distances.

DCA occupies the volume close to the protein surface, clearly promoting a depletion of Cl anions. Furthermore, the MDDF representing the distribution of EMIM cations displays a prominent peak at approximately ∼2.4 Å that indicates dispersive interactions established by EMIM with the protein. Furthermore, accumulation of EMIM occurs in complementary distances than those of DCA, because the ions locally counteract each other charges, in a cooperative way, as shown in a previous study.^43,45^ In summary, DCA exhibits notably more favorable interactions with the protein surface than Cl, both specifically at hydrogen-bonding distances and nonspecifically, by forming a second solvation shell mediated by the accumulation of the cation.

**Figure 3:**
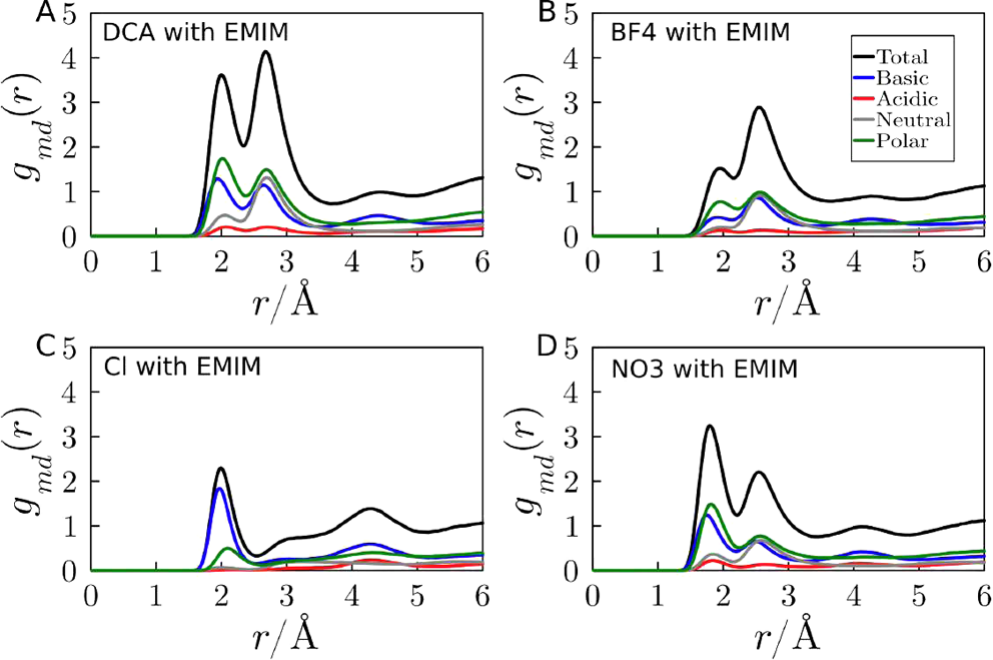
Decomposition of the MDDFs of (A) DCA, (B) BF_4_, (C) Cl, and (D) NO_3_ in the contribution of protein amino acid residue types in systems with IL composed of the cation EMIM and a single type of anion, at 2.0 mol L^–1^. Supporting Information Table S3 contains the classification category of each residue type, which follows that of the visual molecular dynamics (VMD) software.^56^ The total MDDF is the sum of the contributions of each type of residue.

Figure 3 displays the MDDFs of the anions relative to the protein in the IL solutions with a single type of anion. The MDDFs are decomposed in the contributions of the different protein amino acid residue types (the decomposition of the MDDFs into solute and solvent groups is a feature of the ComplexMixtures.jl package). For DCA, BF_4_, and NO_3_, there are distinctive peaks centered around 1.8 and 2.0 Å, which correspond to the hydrogen bonds that these ions establish with the protein. Chlorine displays strong interactions with basic residues, as expected, at slightly larger distances. The first peak for DCA, NO_3_, and BF_4_ is primarily due to interactions with polar and basic residues (which might be, respectively, glutamine and lysine), with which they can form electrostatic interactions and hydrogen bonds. The second peak, which appears between 2.4 and 2.6 Å for these three anions, has additional significant contributions originating from neutral residues (most likely glycine, leucine, and valine). This second peak is probably an interaction mediated by the IL cation, which displays favorable hydrophobic interactions and attracts the anion to the proximity of neutral residues.

**Figure 4:**
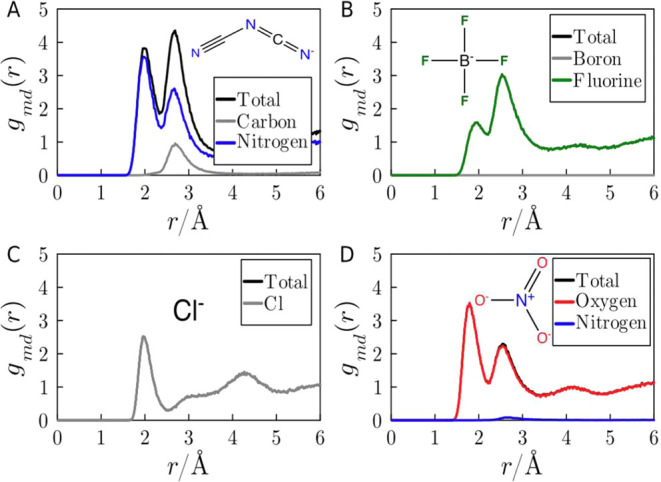
MDDFs of (A) DCA, (B) BF_4_, (C) Cl, and (D) NO_3_, relative to the protein, at 2.0 mol L^–1^ IL solutions. These are the same distributions shown in Figure 3, but now decomposed in the contributions of the atom types of the anions.

Figure 4 displays the same MDDFs as Figure 3, but now with their decomposition in terms of the types of atoms of the anions. DCA has a notable capacity for establishing H-bonds with protein surface atoms through its nitrogen atoms. In contrast, the second peak has important contributions of the Carbon atom, supporting that these interactions are nonspecific. BF_4_ has strong polar interactions (which cannot be associated with H-bonds) and has a prominent peak of nonspecific interactions. Chlorine displays a clear peak at short distances associated with electrostatic bonds with basic residues. And, finally, NO_3_, as expected, forms strong H-bonds through its oxygen atoms.

Table 2 shows the number of anions within a 5.0 Å from the protein surface. Consistent with the insights gained from Figure 2, it is evident that DCA ions are more abundant in the proximity of the protein compared to other anions, particularly relative to Cl ions. NO_3_ follows DCA in its affinity to the surface of the protein. When these anions are present alongside those with a lower tendency to interact closely with the protein, particularly Cl, they collectively constitute the majority of negatively charged entities near the protein.

**Table 2:** Protein Coordination Numbers by EMIM and the Anions, by Counting the Number of Ions up to 5 Å from the Protein Surface, in the Systems with 2.0 mol L^–1^ of IL Mixture^a^

ionic liquid mixture (EMIM + anions)	EMIM	anion 1	anion 2
DCA and BF_4_	47 ± 1	25 ± 1 (DCA)	11 ± 1 (BF_4_)
DCA and NO_3_	47 ± 1	23 ± 1 (DCA)	14 ± 1 (NO_3_)
DCA and Cl	51 ± 1	30 ± 1 (DCA)	7 ± 1 (Cl)
BF_4_ and NO_3_	43 ± 1	14 ± 1 (BF_4_)	18 ± 1 (NO_3_)
BF_4_ and Cl	42 ± 1	18 ± 1 (BF_4_)	12 ± 1 (Cl)
NO_3_ and Cl	44 ± 1	20 ± 1 (NO_3_)	9 ± 1 (Cl)

a The system has 1004 cations and 502 of each anion at this concentration. The fluctuations reported are the standard error of the mean of the 20 replicas performed for each system.

DCA is the anion that most strongly promotes the approach of the cation EMIM to the surface of the protein. A number of EMIM ions between 47 and 51 are found in solutions where DCA is present, while EMIM ions within 5.0 Å without DCA were at most 43. The lower concentration of EMIM cations at this distance from the protein occurs for the solution with BF_4_ and Cl, which are the less affine anions to the protein surface. Clearly, anions with strong interactions with the protein tend to accumulate closer to the protein surface, resulting in a negative net charge, leading by electrostatic compensation to the accumulation of cations.

Both DCA and NO_3_ can form hydrogen bonds with the protein surface, but DCA displays a greater net affinity to it. This can be seen by the greater number of DCA anions in close contact relative to NO_3_ ions. BF_4_ and Cl are less present in the proximity of the protein surface.

**Figure 5:**
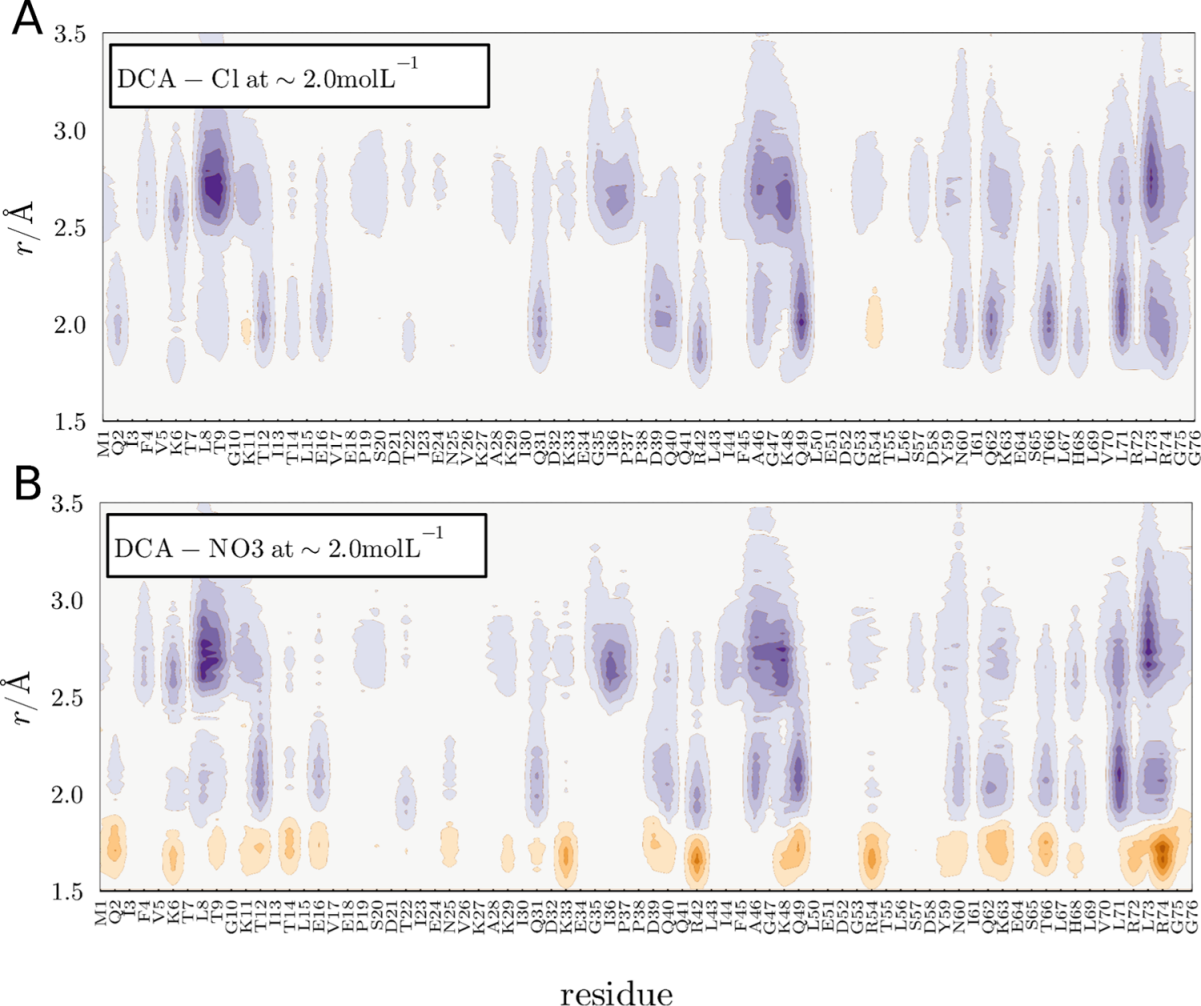
(A) Difference in MDDF densities of the ions in the vicinity of the protein at ∼2.0 mol L^–1^ of EMIMDCA + EMIMCl. The blue purple represents regions where DCA has greater density than Cl, and the orange color represents regions where DCA has a lower density than Cl. (B) Similar profile for the EMIMDCA + EMIMNO_3_ mixture, with purple colors representing, similarly, greater DCA density.

Figure 5 presents differential ion density maps around individual protein residues, in the IL with mixtures of anions. In Figure 5A purple regions indicate a greater density of DCA relative to Cl, and in orange a greater density of Cl relative to DCA. DCA is essentially more concentrated near the protein everywhere relative to Cl. The Chloride ion interacts mainly with basic residues (which have positive charges), as shown in Supporting Information Figure S6, but this interaction is weaker than DCA hydrogen bonding. In Figure 5B, we show the difference in density maps of DCA and NO_3_ in the system with both anions. This map is particularly interesting, because it indicates that NO_3_ displays greater densities at short-ranged hydrogen-bonding distances, while DCA is overall more concentrated at every other solvation distance. The anions compete for hydrogen bonds in the same protein residues and, naively, one would interpret that NO_3_ had a greater affinity than DCA, from the distance distribution of these hydrogen bonds. However, DCA ends up displaying much greater affinity, as evidenced by distribution integrals and preferential interaction parameters, discussed later here.

**Figure 6:**
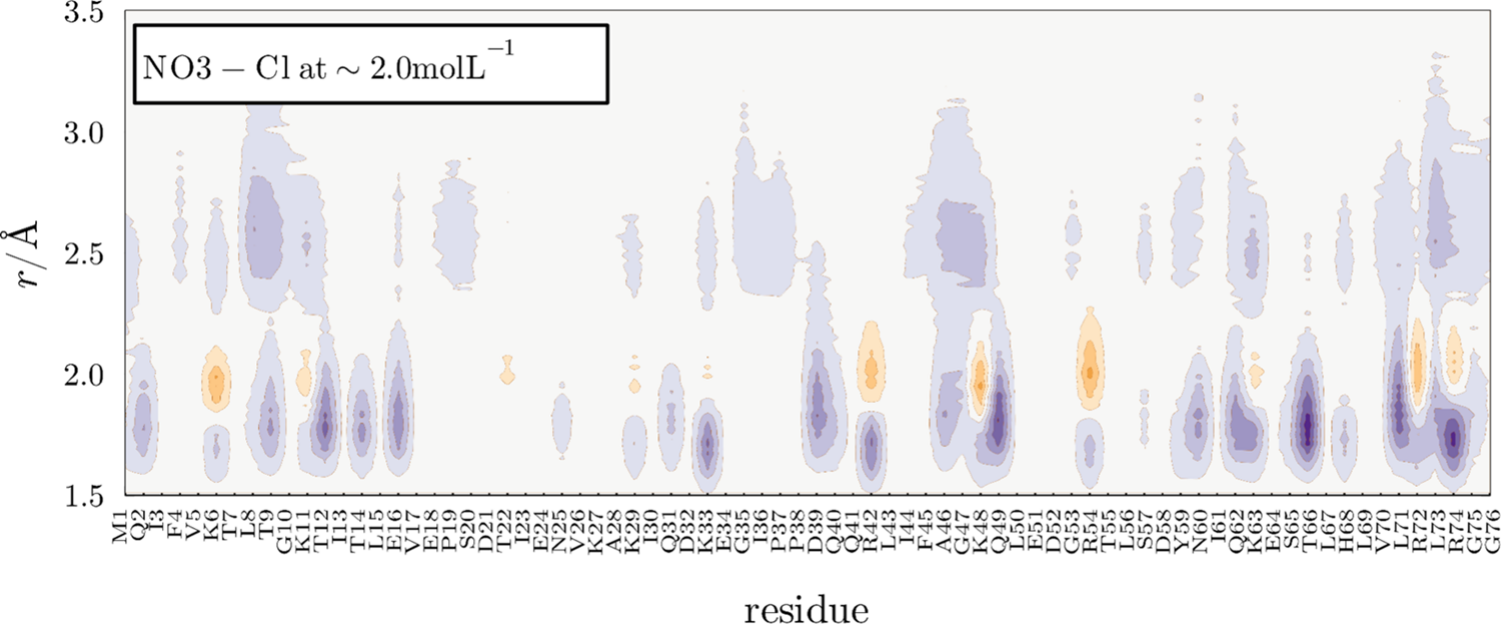
Competition between NO_3_ and Cl for the protein surface. NO_3_ forms hydrogen bonds, and dominates the shortest distances to the protein. However, Cl competes effectively for interactions with positively charged residues and is found preferentially at distances slightly larger than those of H-bonds around those residues. The purple color indicates greater NO_3_ density, and orange indicates greater Cl density.

The Nitrate anion, on the other side, in solutions with Chloride, is not found at a greater concentration than Cl at all distances, as shown in Figure 6. NO_3_ can form hydrogen bonds, which are found at distances shorter than 2.0. However, in the vicinity of basic residues, with positive charge, Cl ions can compete with NO_3_ and be found at greater local density. Thus, despite being a hydrogen-bonding anion, NO_3_ comparative affinity to the protein surface is not enough to match the Cl electrostatic interactions, differently to what is observed for DCA.

The relative affinities of the ions for the protein can be observed from the resulting bulk concentrations in the simulations, after equilibration. For example, in the systems where DCA and Cl should be present in a 1 mol L^–1^ concentration each, the resulting bulk concentration for DCA was 0.98 mol L^–1^ and that of Cl 1.10 mol L^–1^, illustrating that DCA is effectively accumulated on the protein domain to a greater extent. Similarly, in the system with DCA and NO_3_, the equilibrium bulk concentrations were 1.03 and 1.06 mol L^–1^, respectively, thus while DCA is found at a lower concentration in the bulk, the difference relative to NO_3_ is smaller and the divergence relative to the target concentration are also smaller.

### Effect of Multiple Anions on the Kirkwood–Buff Integrals

**Figure 7:**
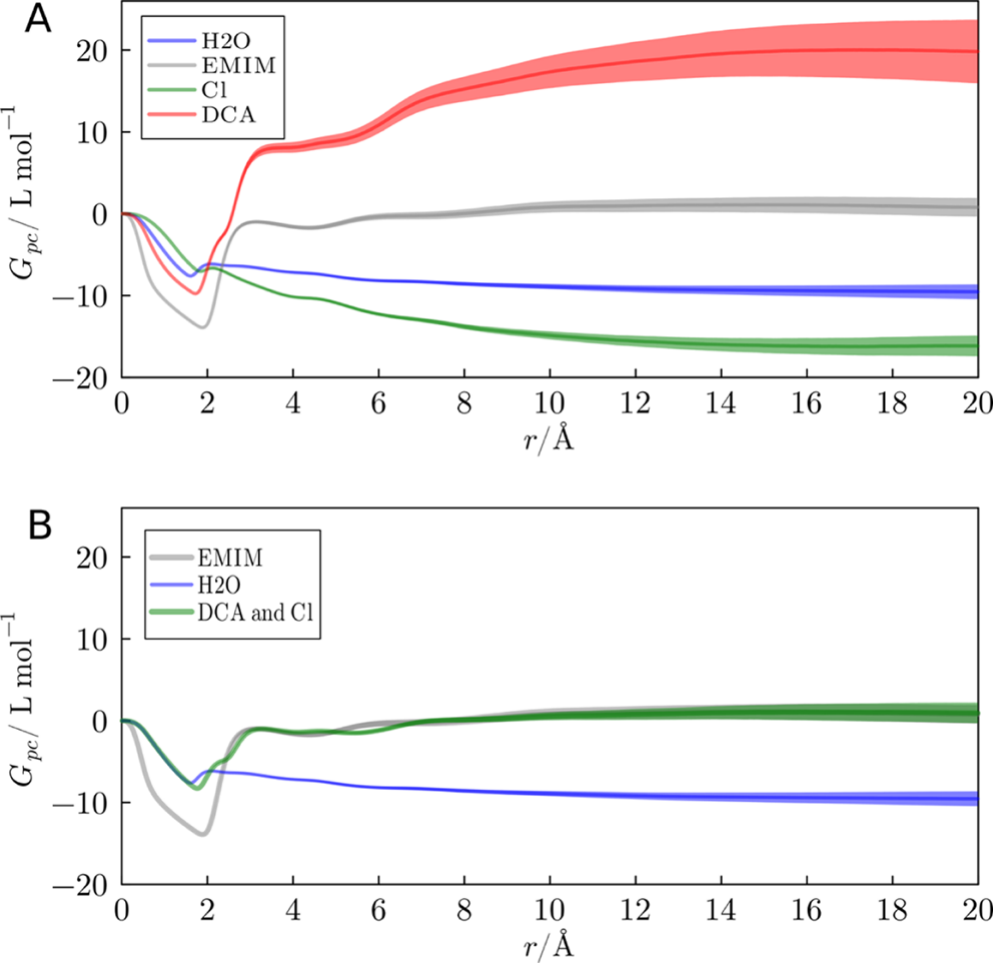
(A) KB integrals of water, EMIM, Cl, and DCA relative to the protein in systems with 2.0 mol L^–1^ of IL mixture. Solid lines are mean values of 20 simulation replicas, and shades represent the standard error of the mean. (B) Ion KB integrals (EMIM and DCA + Cl are overlapped) compared to water integrals. The greater KB integrals for ions relative to water shows that the protein is preferentially solvated by the IL ions.

The KB integrals provide insights into the effective accumulation or depletion of solvent molecules in the protein domain. In Figure 7, we present the KB integrals for ions and water in a system with a 2.0 mol L^–1^ concentration. The quick drop at short distances (*r* < 1.5 Å) is associated with the exclusion volume imposed by the protein. This initial descent is succeeded by an accumulation phase encompassing specific and nonspecific direct solute–solvent interactions, occurring in the 1.9–5.0 Å range. The KB integral for DCA converges to a positive value, which indicates the strong interactions of this ion with the protein, implying an accumulation on the protein domain that is enough to counteract the exclusion volume. The KB integral for EMIM in this system is close to zero, and the Chloride and water integrals are negative.

As anticipated by the depletion of Chloride ions in the MDDF of Figure 2, the KB integral for Cl is negative, indicating that Cl is excluded from the protein domain in the presence of DCA. EMIM, the sole source of positive charge, has a KB integral value intermediate to those DCA and Cl. A KB integral value of zero signifies that the compound is neither accumulated nor depleted in the protein domain. In Figure 7B, the KB integrals consider both anions as indistinguishable entities. As expected, the KB integrals for cations and all anions are equal because the bulk solution must be neutral, such that the number of cations and anions in the protein domain must be the same.^43−45^

**Figure 8:**
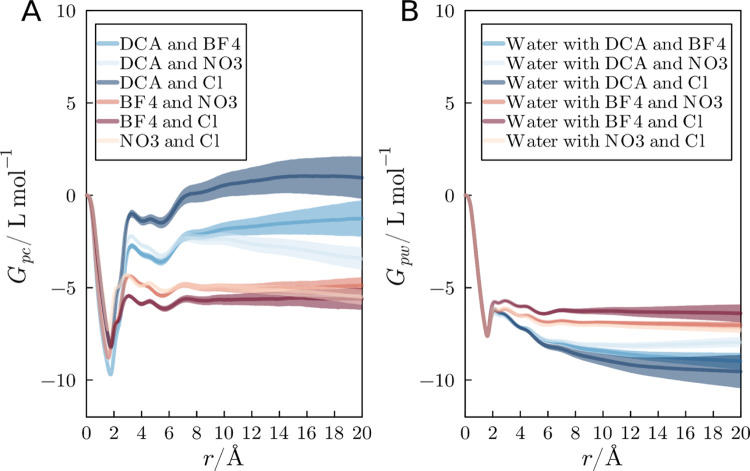
(A) KB integrals considering the anions as one entity and (B) water KB integrals in systems with 2.0 mol L^–1^ of IL mixture. The mixture containing DCA anions exhibits significantly higher KB integral values compared to the other systems. Notably, the combination of DCA with anions displaying a lower tendency to interact with protein surface atoms results in the highest KB integral values, highlighting the enhanced accumulation effects in this scenario.

Figure 8A illustrates the total cation or anion KB integrals relative to the protein for all the systems simulated with 2.0 mol L^–1^ IL mixtures. Figure 8B displays the corresponding integrals for water in the same systems. As previously highlighted, DCA exhibits the highest propensity to accumulate in the vicinity of the protein, and thus the systems with DCA display the greater ion integrals, and the smaller water KB integrals. Combining a high-affinity anion (DCA) with a low-affinity one (Cl) results in an overall greater accumulation of DCA, and anions in general, in the protein domain. The increased accumulation of DCA in the DCA + Cl mixture results in a greater DCA density in the protein domain and, frequently, in a greater overall preferential interaction parameters of the IL relative to water, as it will be shown.

Water KB integrals exhibit negative values in the systems depicted in Figure 8B. This indicates a substantial depletion of water molecules compared to their distribution in the bulk environment. The trend of KB values is the opposite to that of that observed for the anions. Consequently, in systems with larger KB integrals for anions, water molecules are more effectively excluded compared to those systems where the anions’ KB integrals are less significant.

### Effect of Multiple Anions on Preferential Solvation

The preferential solvation parameter (Γ) is a valuable tool for examining how a cosolvent interacts and affects macromolecules’ structural stability.^57,58^ Essentially, the preferential solvation parameter reflects the change in the protein chemical potential due to adding cosolvents to the system.^57^ When Γ is positive, the cosolvent interacts favorably with the protein’s surface, stabilizing structures with larger surface areas, often associated with denatured states. Conversely, if the protein is preferentially hydrated, the cosolvent is repelled from the protein’s surface, promoting the formation of more compact structures typically associated with folded and functional protein conformations. This fundamental concept forms the basis for understanding the overall stabilizing or destabilizing impact of osmolytes on protein structures.^57,58^

Preferential solvation parameters are computed from the difference between KB integrals of the components of the solvent using
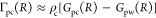
2where the subscripts pc and pw refer to protein-cosolvent (the IL) and protein–water.^59−62^ If Γ_pc_(*R*) is positive (the KB integral of the IL is greater than that of water), the IL preferentially solvates the protein. Here, Ubiqutin is neutral, but for proteins with a nonzero net charge, preferential solvation parameters change, adapting the formula for Γ_pc_(*R*) o account for ionic releases during solvation, incorporating a corrective parameter and the biomolecule’s absolute charge as detailed by Pierce and colleagues.^59^

**Figure 9:**
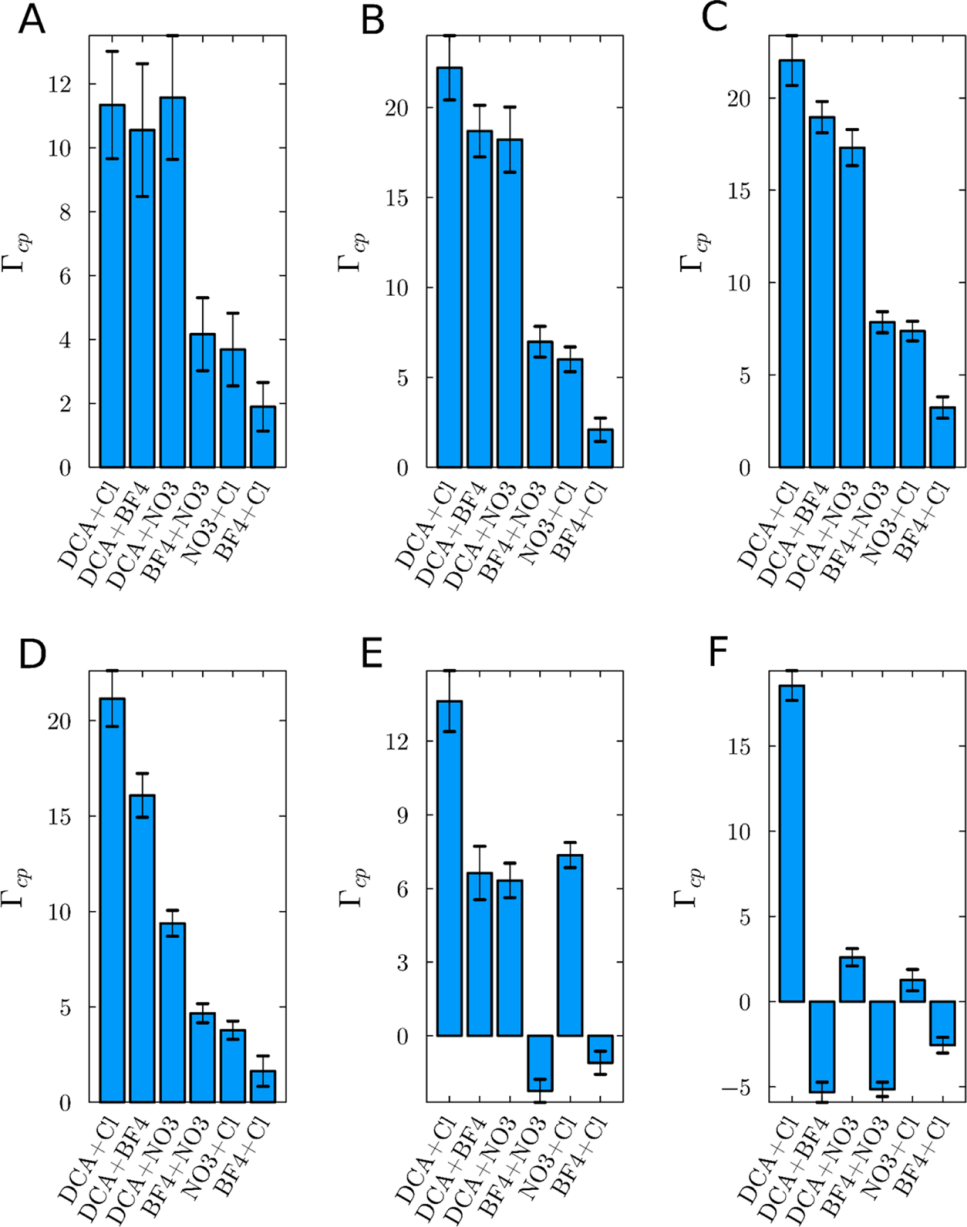
Preferential solvation parameters for the IL relative to the protein in all concentrations simulated with different anion compositions: with (A) 0.5, (B) 1.0, (C) 1.5, (D) 2.0, (E) 2.5, and (F) 3.0 mol L^–1^ solutions, providing a comparative analysis of IL behavior across varying solute concentrations. The chart highlights the ions’ varying tendencies to preferentially solvate the protein over water in the presence of varying competing ions. Because of the electroneutrality of the solution, these parameters are identical to those computed for the cation or the set of anions in each solution.^43,45^

Figure 9 illustrates the preferential solvation parameter for the ions relative to the protein across the range of concentration simulated, ranging from 0.5 to 3.0 mol L^–1^ ILs. The preferential interaction parameters are mostly positive in systems with the anion combinations of DCA + Cl, DCA + NO_3_, and DCA + BF_4_, especially 0.5 up to 2.0 mol L^–1^. Similarly, the combinations of BF_4_ + NO_3_ and NO_3_ + Cl also display positive values. This indicates that in most cases where the IL preferential solvation is positive, the ILs tend to reduce water’s affinity for the protein surface.^63,64^ Consequently, these ILs are expected to act as denaturants, promoting protein conformations with larger surface areas. The systems with DCA show the most significant reduction in water interaction, notably DCA + Cl, DCA + BF_4_, and DCA + NO_3_, in this order. The combination of DCA + Cl leads to the highest values for the preferential solvation parameter in most cases, as illustrated in Figure 9. However, despite the trend of DCA + Cl resulting in the greatest solvation, it is noteworthy that at a concentration of 0.5 mol L^–1^, the values for combinations involving DCA are statistically equivalent when considering the standard error calculated from the 20 simulations. Although the specific effects of different anion combinations are not entirely clear, those involving the DCA anion particularly result in greater protein dehydration.

**Figure 10:**
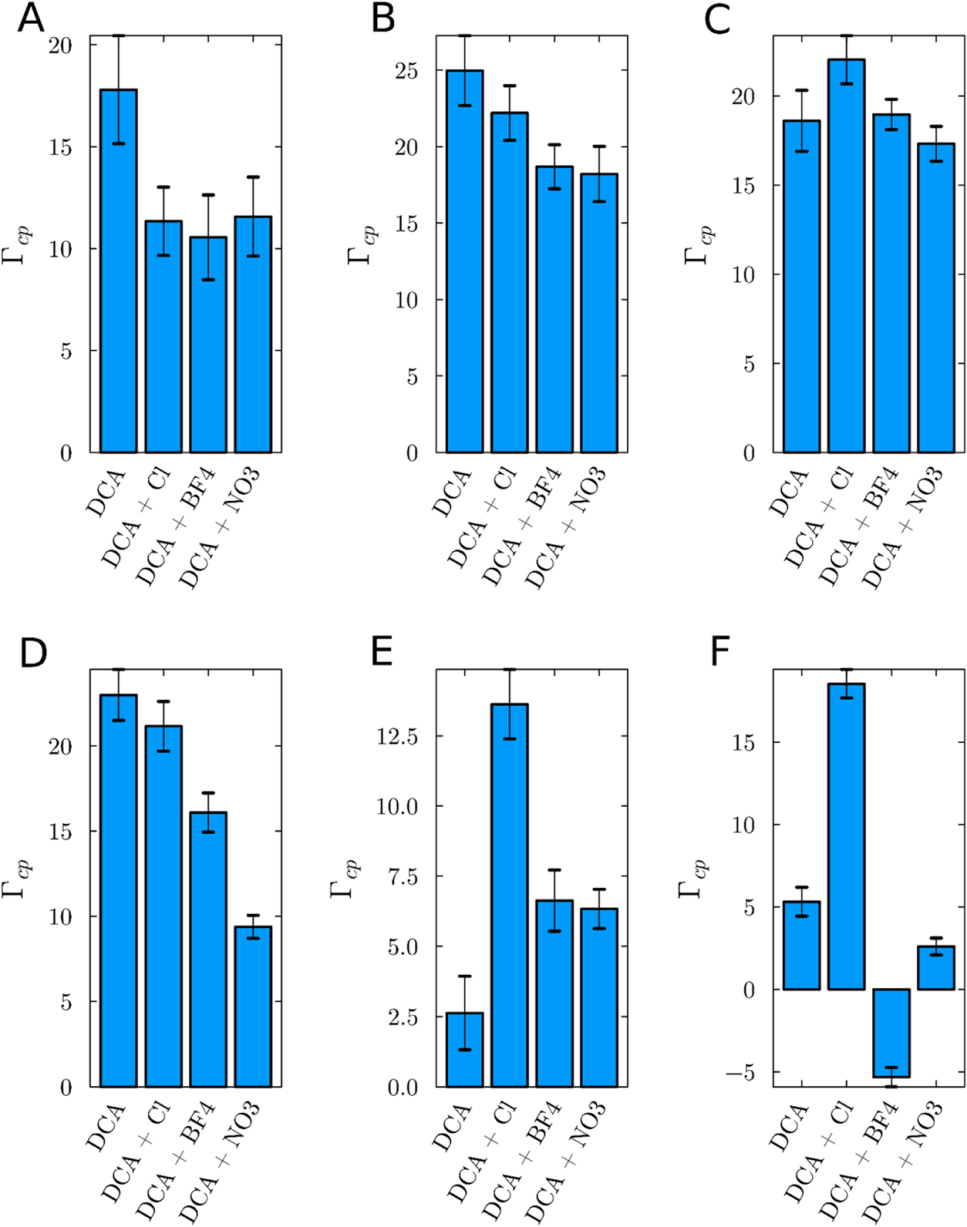
Analysis of IL preferential solvation parameters (Γ_cp_) comparing a system containing only DCA to systems where DCA is mixed with Cl, NO_3_ and BF_4_ at concentrations of (A) 0.5, (B) 1.0, (C) 1.5, (D) 2.0, (E) 2.5, and (F) 3.0 mol L^–1^. This comparative analysis highlights the behavior of ILs across a range of solute concentrations. Error bars indicate the standard error of the mean of 20 replicas for each concentration.

Figure 9 illustrates that certain ion combinations exhibit greater preferential solvation parameters than others, notably those including the DCA anion. While combinations such as DCA + Cl, DCA + NO_3_, and DCA + BF_4_ demonstrate significant ion accumulation compared to other mixed systems, their accumulation levels are generally lower than those observed in systems exclusively containing DCA across most concentrations. Figure 10 displays the preferential solvation parameters in solutions of ILs containing DCA only and mixtures of DCA with the other anions. The Supporting Information Figure S7 provides a comprehensive overview of the preferential solvation parameters for all other single-IL systems.

Figure 10 predominantly indicates that the preferential solvation values for the IL are higher in the system with pure EMIMDCA at lower concentrations. At higher concentrations the trends oscillate, and the DCA+Cl combination exhibits higher preferential solvation than the DCA-only system. However, at higher concentrations, shown in Figure 10E,F, the trends observed can be attributed to instabilities in the computation of the preferential solvation parameter. Specifically, Figure S10 in the Supporting Information indicates that certain Kirkwood-Buff integrals for water fail to converge properly at high IL concentrations, resulting in variations in the preferential parameter that do not align with the trends observed in the lower concentrations simulated.

A careful examination of Figure 9 shows that for the smaller concentrations (0.5 to 2.0 mol L^–1^) general trends for the preferential interaction parameters can be obtained, and are not trivial to interpret. We know that the anion affinity to the protein follows the sequence: DCA > NO_3_ > BF_4_ > Cl (Supporting Information Figure S7). If the contributions of the anions to the affinity of the IL to the protein were additive, combinations of ions would exhibit affinities following a similar trend to that observed for individual anions. The smaller preferential solvation parameters can be associated to more spread ion distributions, as indicated by the MDDFs with lower peaks and less pronounced dips in the KB integrals.

The anion affinity additive nature is effectively observed for the sequences of preferential binding parameters when the preserved cations are NO_3_, BF_4_, and Cl, in the concentration range up to 2.0 mol L^–1^. For instance, in the case of NO_3_, the preferential binding trend for the mixtures is NO_3_ + DCA > NO_3_ + BF_4_ > NO_3_ + Cl (Figure 9D–F—noteworthy at 2.0 mol L^–1^). The same additive nature is observed for the sequence of preferential interaction parameters with the common ion being BF_4_ or Cl.

However, for DCA, the preferential solvation reveals a contrasting trend in protein affinity: DCA + Cl > DCA + BF_4_ > DCA + NO_3_. This inversion of order, as compared to NO_3_, BF_4_, and Cl, is intriguing, and reflects a nonadditive affinity behavior when DCA is present. To understand how the combination of DCA with lower affinity ions has a smaller impact in the IL preferential binding to protein, we speculate that an adsorption model including both cooperative and competitive effects and multiple types of binding sites is necessary, and will be the subject of future research. In any case, the differences in bulk solvation of the ions (Supporting Information Tables S5 and S6) do not seem to justify these differences.

In summary, these results illustrate that preferential interactions and the molecular properties of the solvation of complex solutes, as proteins, cannot be deduced trivially from the combinations of the binding characteristics of each component of the mixtures, illustrating the general difficulty of interpreting solvation thermodynamics from the structure of the species involved.^5,65^ The interactions between ions and proteins are difficult to generalize or model from reduced or simplified assumptions about the nature of the ions involved. Moreover, the specific nature of the interactions between the ions and the solute is pivotal in determining the overall properties of the solution.^7^ For instance, it is known that the Hofmeister series does not accurately predict the effects of certain salts on lysozyme structure.^7,66^ The salting-out effect, as forecasted by the Hofmeister series, occurs predominantly at basic pH levels and under high ionic strength conditions. However, this effect deviates significantly from Hofmeister series predictions under neutral and acidic conditions, illustrating a more complex interaction pattern than previously understood.^66−68^

## Conclusions

In this study, we have explored the solvation structure of proteins in the presence of various ionic liquids (ILs), specifically focusing on those composed of the EMIM cation combined with an array of anions possessing distinct chemical properties. We identified specific interactions as pivotal in driving the enhanced preferential solvation observed, particularly in systems with DCA and NO_3_ anions. It was noted that DCA exhibits a significantly greater tendency to form hydrogen bonds with proteins compared to other anions, being this formation of stable interactions likely determinant for a distinct behavior. However, this propensity is attenuated by the presence of competing ions, underscoring a competitive effect that is evident upon analyzing the preferential solvation parameters. These parameters generally decrease for mixtures relative to DCA-only systems, suggesting a nuanced competitive interaction among anions. Nevertheless, the impact of weaker binding anions on solvation properties remains less clear, indicating a rich avenue for further investigation. Future research should focus on dissecting the roles of individual anions within mixed IL solutions, employing a combination of experimental and theoretical approaches to unravel the complex interplay of forces at play.

## Supporting Information Available

Post-NPT concentrations of all components in bulk (Table S1); number of components for each simulation box (Table S2); criteria for classifying residues as acidic, basic, neutral, and polar (Table S3); additional information on MDDFs and KBIs for all systems (Figures S1–S5 and S10); density distribution 2D map of chloride in systems with DCA (Figure S6); data on preferential solvation parameters for single ionic liquid systems (Figure S7); protein RMSD for all systems (Figure S8); water coordination number for mixed ionic liquid systems (Figure S9); interaction energy decomposition into Lennard–Jones and Coulomb energies (Table S4); additional preferential solvation parameter data for all systems (Tables S5 and S6) (PDF)
