# Supplementary material for: Competitive Effects of Anions on Protein Solvation by Aqueous Ionic Liquids
Source: J Phys Chem B. 2024 Aug 2;128(32):7792–802. doi: 10.1021/acs.jpcb.4c03735 (PMC11331513; doi:10.1021/acs.jpcb.4c03735)
Supplement: Supplementary file 1 — jp4c03735_si_001.pdf [file jp4c03735_si_001.pdf]

Supporting Information

Competitive effects of anions on protein solvation by aqueous ionic liquids

Vinicius Piccoli[1] and Leandro Martínez[1,]*

Institute of Chemistry and Center for Computing in Engineering & Science, Universidade Estadual de Campinas (UNICAMP), 13083-861 Campinas, SP, Brazil

---

Corresponding Author:

*Leandro Martínez: lmartine@unicamp.br, Institute of Chemistry, Universidade Estadual de Campinas (UNICAMP). 13083-970, Campinas, SP. Brazil. http://m3g.iqm.unicamp.br

Table S1. Reference concentrations (RC) of ionic liquids (IL) and bulk concentrations of components Post-NPT equilibration across all simulated systems. The table details fluctuations calculated through the standard error of the mean, derived from each concentration's 20 simulations.

Ionic Liquid	RC (mol/L)	Water (mol/L)	Cation (mol/L)	Anion1 (mol/L)	Anion 2 (mol/L)
EMIMDCA + EMIMBF4	0.50	50.55 ± 0.02	0.48 ± 0.02	0.23 ± 0.02	0.25 ± 0.01
	1.00	46.26 ± 0.02	0.98 ± 0.03	0.47 ± 0.03	0.51 ± 0.02
	1.50	41.68 ± 0.02	1.52 ± 0.03	0.74 ± 0.02	0.78 ± 0.01
	2.00	36.85 ± 0.04	2.08 ± 0.04	1.02 ± 0.03	1.063 ± 0.01
	2.50	31.83 ± 0.04	2.67 ± 0.05	1.32 ± 0.05	1.35 ± 0.03
	3.00	26.63 ± 0.02	3.28 ± 0.03	1.63 ± 0.04	1.66 ± 0.03
EMIMBF4 + EMIMNO3	0.50	50.718 ± 0.008	0.497 ± 0.001	0.249 ± 0.0	0.249 ± 0.0
	1.00	46.611 ± 0.012	1.016 ± 0.002	0.512 ± 0.001	0.501 ± 0.001
	1.50	42.298 ± 0.013	1.56 ± 0.02	0.786 ± 0.002	0.776 ± 0.002
	2.00	37.708 ± 0.015	2.11 ± 0.02	1.077 ± 0.001	1.066 ± 0.002
	2.50	32.903 ± 0.018	2.75 ± 0.02	1.384 ± 0.002	1.367 ± 0.002
	3.00	27.936 ± 0.017	3.387 ± 0.003	1.708 ± 0.003	1.681 ± 0.004
	0.50	50.889 ± 0.013	0.48 ± 0.03	0.233 ± 0.001	0.249 ± 0.0

	1.00	46.908 ± 0.029	0.985 ± 0.003	0.477 ± 0.004	0.505 ± 0.002
**EMIMDCA + EMIMNO3**	1.50	42.672 ± 0.024	1.521 ± 0.003	0.746 ± 0.003	0.773 ± 0.002
	2.00	38.158 ± 0.022	2.09 ± 0.003	1.036 ± 0.003	1.056 ± 0.003
	2.50	33.591 ± 0.026	2.666 ± 0.003	1.324 ± 0.004	1.343 ± 0.003
	3.00	28.863 ± 0.023	3.263 ± 0.003	1.626 ± 0.004	1.638 ± 0.003
	0.50	51.163 ± 0.015	0.48 ± 0.001	0.254 ± 0.001	0.225 ± 0.002
	1.00	46.510 ± 0.031	0.973 ± 0.003	0.517 ± 0.001	0.457 ± 0.004
**EMIMCl + EMIMDCA**	1.50	42.013 ± 0.049	1.500 ± 0.003	0.784 ± 0.001	0.716 ± 0.005
	2.00	38.260 ± 0.089	2.048 ± 0.004	1.061 ± 0.003	0.987 ± 0.007
	2.50	34.512 ± 0.111	2.62 ± 0.01	1.334 ± 0.005	1.287 ± 0.01
	3.00	30.294 ± 0.028	3.19 ± 0.05	1.632 ± 0.005	1.558 ± 0.009
	0.50	51.01 ± 0.01	0.496 ± 0.001	0.252 ± 0.001	0.244 ± 0.001
	1.00	47.051 ± 0.011	1.009 ± 0.001	0.513 ± 0.001	0.497 ± 0.001
**EMIMCl + EMIMNO3**	1.50	43.472 ± 0.017	1.544 ± 0.001	0.785 ± 0.001	0.76 ± 0.002
	2.00	39.613 ± 0.017	2.116 ± 0.002	1.068 ± 0.001	1.049 ± 0.003
	2.50	35.460 ± 0.026	2.695 ± 0.002	1.363 ± 0.002	1.332 ± 0.003
	3.00	31.425 ± 0.016	3.309 ± 0.002	1.664 ± 0.002	1.644 ± 0.003

**EMIMCl + EMIMBF4**	0.50	50.596 ± 0.009	0.504 ± 0.003	0.253 ± 0.002	0.25 ± 0.002
	1.00	46.414 ± 0.014	1.052 ± 0.02	0.528 ± 0.009	0.524 ± 0.01
	1.50	42.027 ± 0.024	1.646 ± 0.05	0.826 ± 0.025	0.82 ± 0.025
	2.00	38.229 ± 0.048	2.322 ± 0.11	1.163 ± 0.052	1.159 ± 0.058
	2.50	33.940 ± 0.119	2.845 ± 0.135	1.408 ± 0.055	1.436 ± 0.08
	3.00	29.400 ± 0.028	3.497 ± 0.173	1.743 ± 0.087	1.753 ± 0.086

Table S2. IL preferential solvation parameters in mixed-IL systems at different concentrations: showcases solvation parameters for ionic liquids (ILs) in six distinct concentrations, with A) 0.5, B) 1.0, C) 1.5, D) 2.0, E) 2.5, and F) 3.0 mol L-1 solutions, providing a comparative analysis of IL behavior across varying solute concentrations. verview of simulation boxes and component counts for initial system setups in all simulated systems. This table presents the dimensions of each simulation box, determined post-NPT equilibration, along with the number of components used to construct the initial systems.

Ionic Liquid Mixture	RC (mol/L)	Equilibrated Box side (Å)	Number of cations	Number of anions of type 1	Number of anions of type 2	Number of water molecules
EMIMDCA + EMIMBF4	0.50	94.6	252	126	126	25300
	1.00	94.1	502	251	251	22695
	1.50	93.5	752	376	376	20090
	2.00	93.0	1004	502	502	17463
	2.50	92.4	1254	627	627	14858
	3.00	91.8	1504	752	752	12253
EMIMBF4 + EMIMNO3	050	94.3	252	126	126	225334
	100	93.7	502	251	251	22761
	150	93.1	752	376	376	20188
	200	92.4	1004	502	502	17595
	250	91.7	1254	627	627	15023

	300	91.0	1504	752	752	12540
**EMIMDCA+ EMIMNO3**	050	94.6	252	126	126	25474
	100	94.0	502	251	251	23041
	150	93.4	752	376	376	20607
	200	92.9	1004	502	502	18155
	250	92.4	1254	627	627	15721
	300	91.8	1504	752	752	13288
**EMIMCl + EMIMDCA**	050	94.7	252	126	126	25659
	100	94.2	502	251	251	23410
	150	93.7	752	376	376	21161
	200	93.4	1004	502	502	18894
	250	92.6	1254	627	627	16646
	300	92.0	1504	752	752	14397
**EMIMCl + EMIMNO3**	050	94.5	252	126	126	25687
	100	93.9	502	251	251	23466
	150	93.3	752	376	376	21246
	200	92.7	1004	502	502	19007

	250	92.1	1254	627	627	16786
	300	91.5	1504	752	752	14565
**EMIMCl + EMIMBF4**	050	94.5	252	126	126	25514
	100	93.9	502	251	251	23121
	150	93.2	752	376	376	20728
	200	92.5	1004	502	502	18400
	250	92.1	1254	627	627	15922
	300	91.4	1504	752	752	13529

Table S3. Classification criteria based on Visual Molecular Dynamics (VMD) program. Basic residues are those positively charged due to their ability to accept protons. Acidic residues are characterized by a negative charge resulting from their propensity to donate proteins. Polar residues are those uncharged. Neutral residues are those nonpolar and characterized by their hydrophobic nature, not forming hydrogen bonds or interacting favorably with water. This classification is pivotal as it relies on the intrinsic side chain properties of each residue, particularly their capability to donate or accept protons, thereby dictating their interaction within the molecular environment. During system preparation, we employ the GROMACS tool, pdb2gmx, which automatically assigns protonation states to the residues. This assignment is based on canonical pKa values, with the system's pH assumed to be 7.

Basic	Acidic	Polar	Neutral
This group includes Arginine (R), Lysine (K), and Histidine (H).	Aspartic acid (D) and Glutamic acid (E) fall into this category.	This category encompasses Serine (S), Threonine (T), Asparagine (N), and Glutamine (Q), among others.	Alanine (A), Valine (V), Leucine (L), Isoleucine (I), Methionine (M), Phenylalanine (F), Tryptophan (W), Proline (P), and Glycine (G).

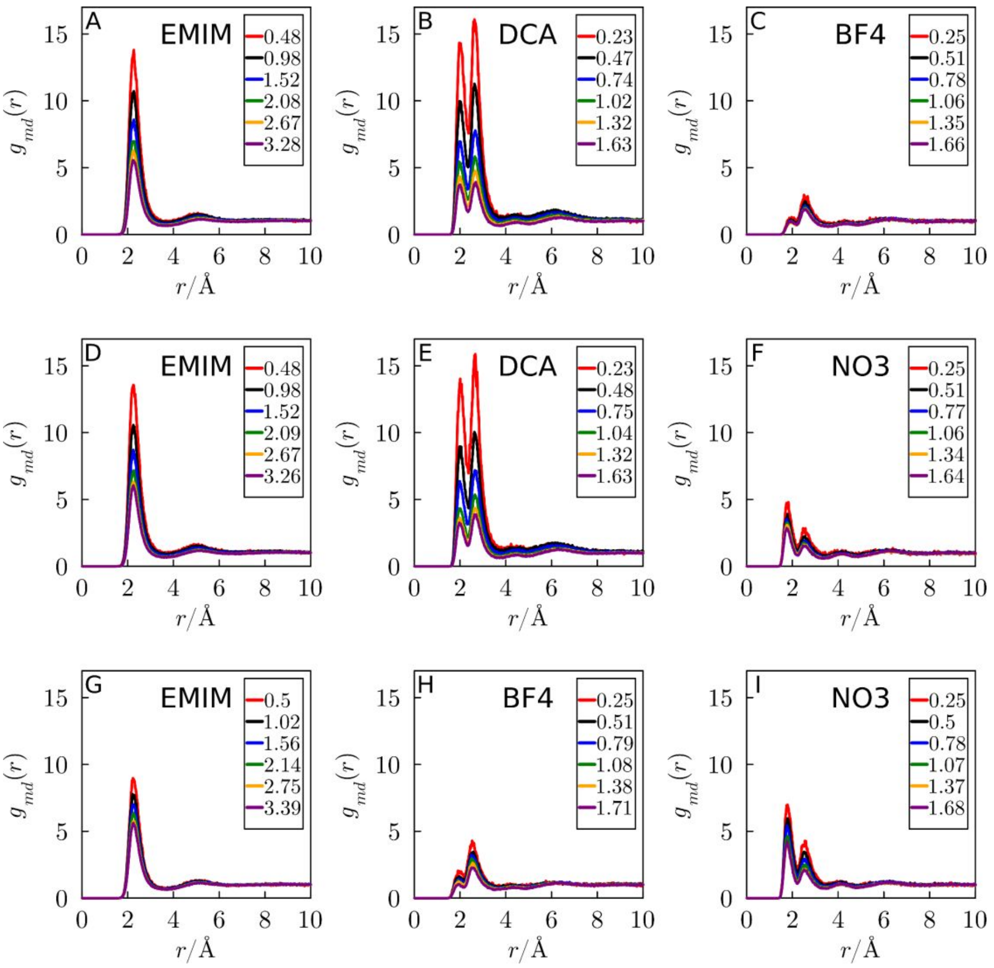

Figure S1. Distribution functions of minimum distances in systems simulated with mixtures of ionic liquids. This figure shows the recalculated concentrations following system equilibration. The curves are displayed up to 10 Angstroms to enhance the visualization of key peaks. Subfigures A), B), and C) represent EMIMDCA + EMIMBF4; D), E), and F) correspond to EMIMDCA + EMIMNO3; and G), H), and I) EMIMBF4 + EMIMNO3.

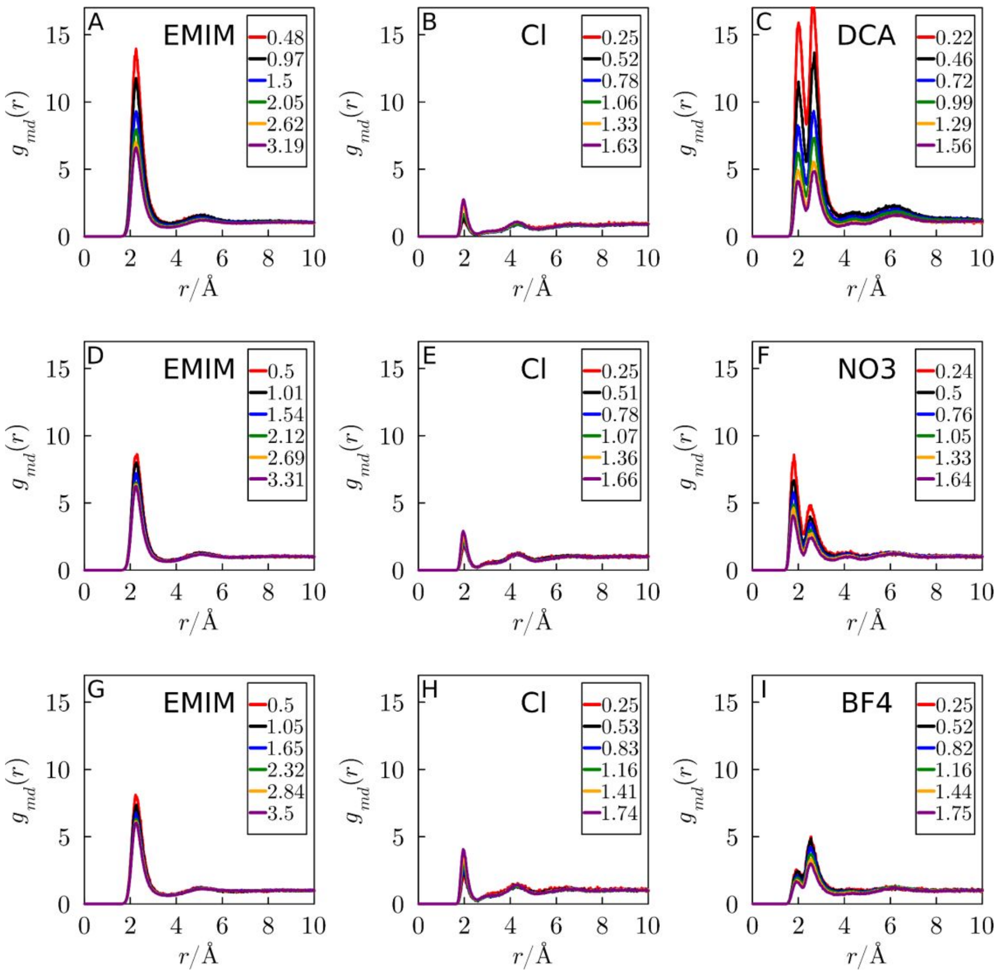

Figure S2. Distribution functions of minimum distances in systems simulated with mixtures of ionic liquids. This figure shows the recalculated concentrations following system equilibration. To enhance visualization of key peaks, the curves are displayed up to 10 Angstroms. Subfigures A), B), and C) represent EMIMCl + EMIMDCA; D), E), and F) correspond to EMIMCl + EMIMNO3; and G), H), and I) EMIMCl + EMIMBF4.

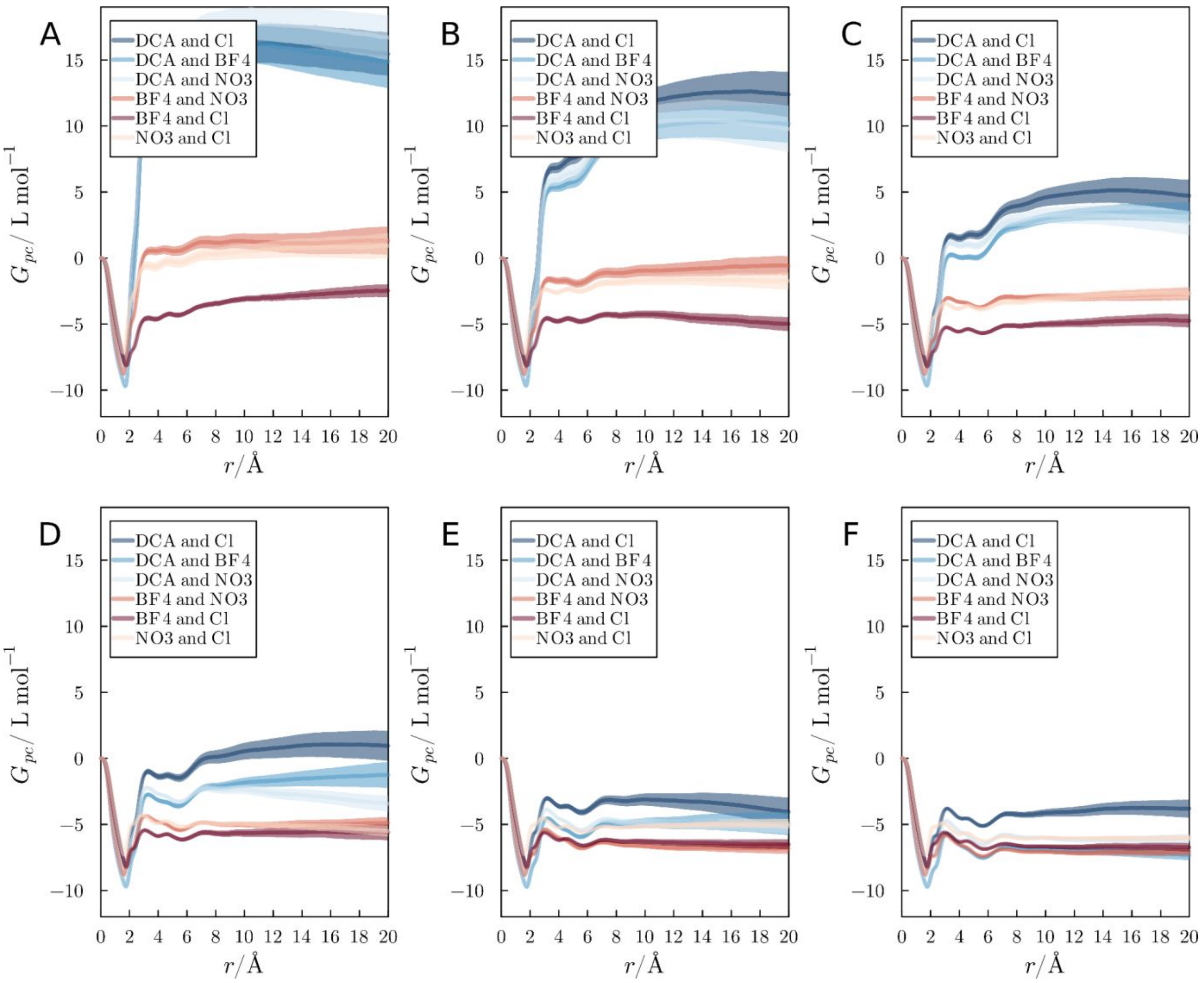

Figure S3. Kirkwood-Buff integrals for all simulated systems with two types of anions. The integrals are calculated over distances that ensure proper convergence for the majority of the systems. Subfigures contain the total KB integral for the anions in systems with A) 0.50, B) 1.00, C) 1.50, D) 2.00, E) 2.50 and F) 3.00 mol L^{-1} IL concentration. The KB integrals for the cations or for the anion pairs converge to the same values, because of the necessary electroneutrality of the bulk solution in the presence of a solute with no net charge.

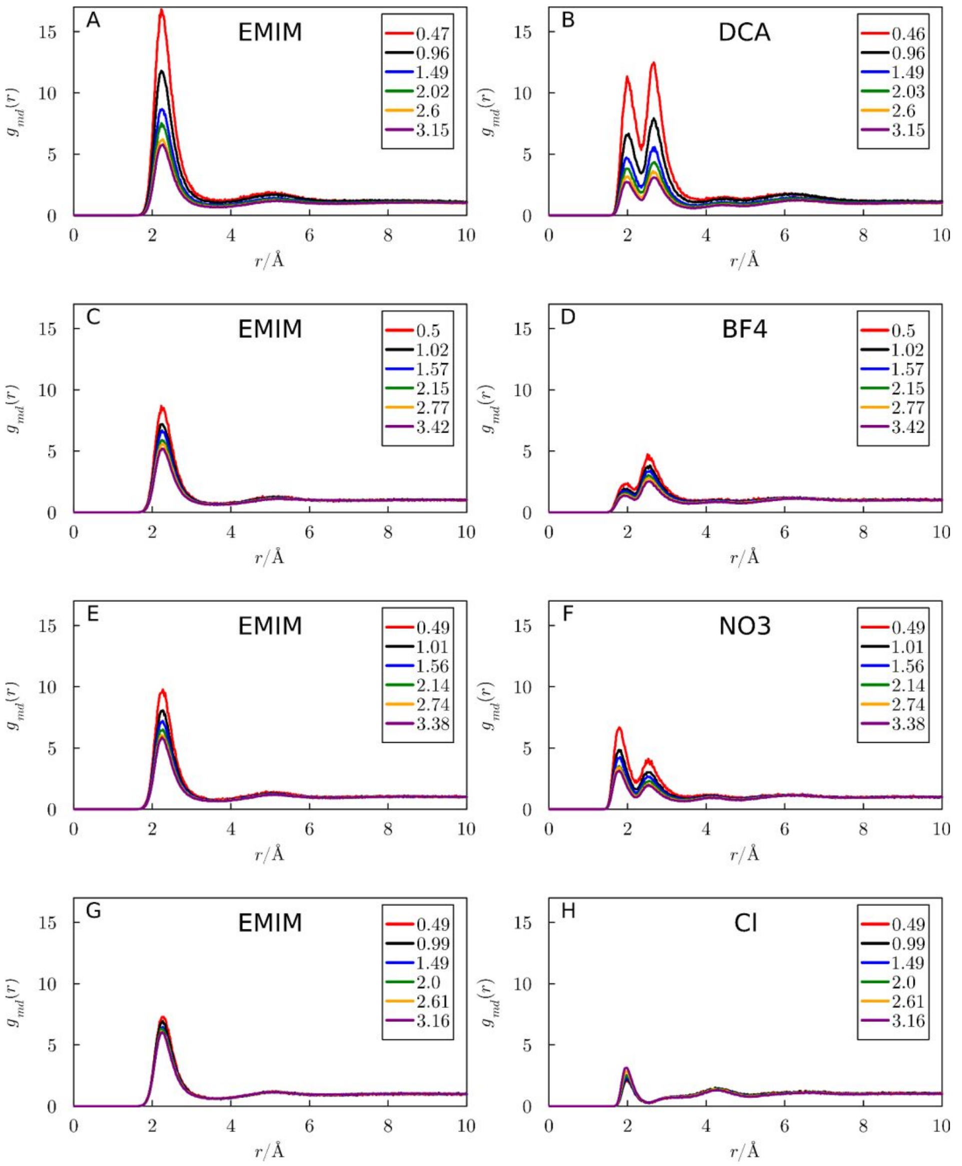

Figure S4. Distribution functions of minimum distances in systems simulated with one ionic liquid. This figure shows the recalculated concentrations following system equilibration. To enhance visualization of key peaks, the curves are displayed up to 10 Angstroms. Subfigures A), represent EMIMDCA; B) EMIMBF4; C) EMIMNO3; D) EMIMCl.

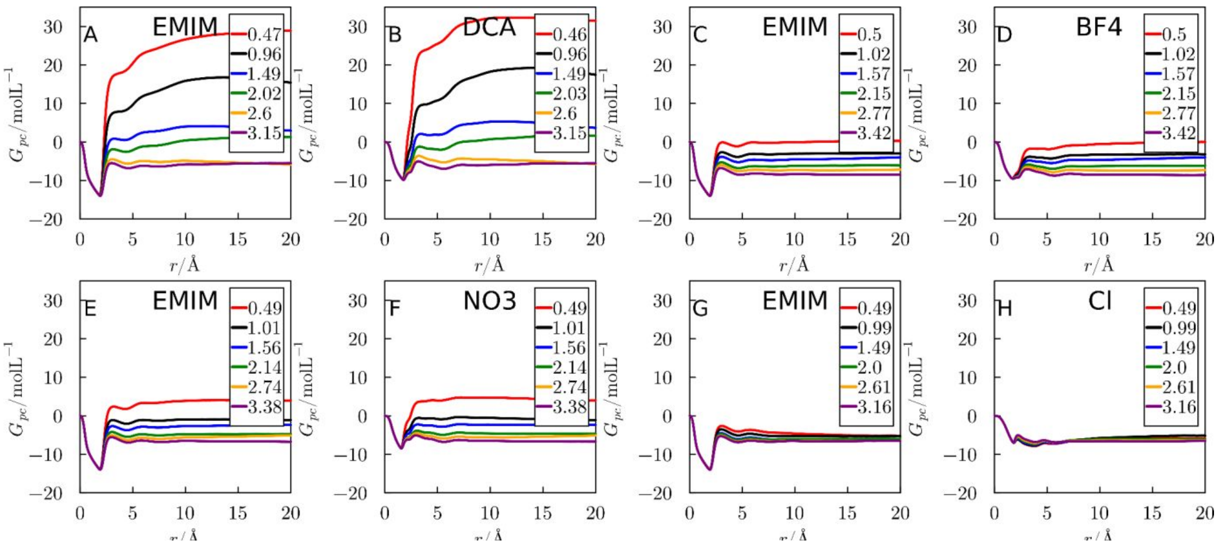

Figure S5. Kirkwood-Buff Integrals for all simulated systems with a single type of anion. The integrals are calculated over lengths that ensure proper convergence for the majority of the systems. Subfigures A) and B), represent EMIMDCA; C) and D), represent EMIMBF4; E) and F), represent EMIMNO3; G) and H) represent EMIMCl.

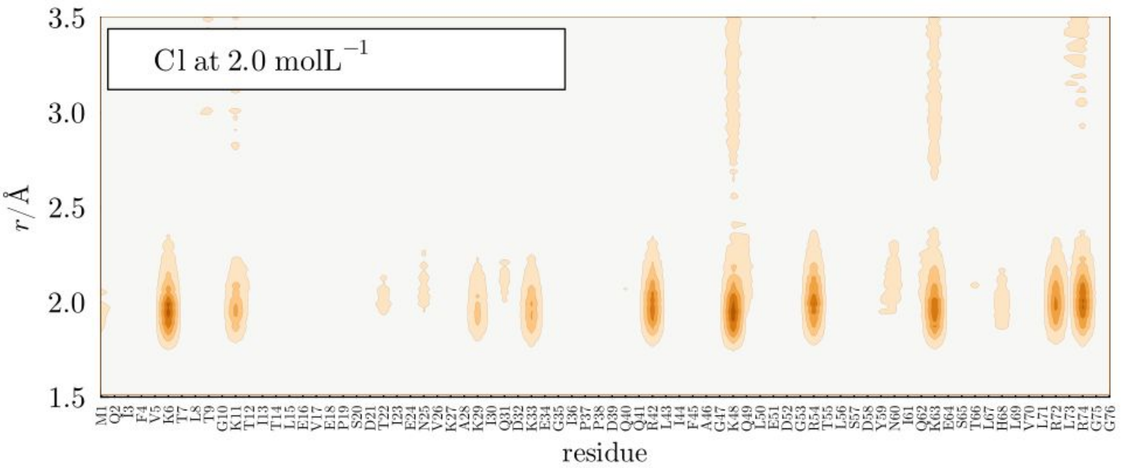

Figure S6. Density of chloride in the 2.0 mol L^{-1} solution containing DCA. Chloride is found to form electrostatic interactions with positively charged residues, as expected, but these interactions are much weaker than DCA hydrogen bonds.

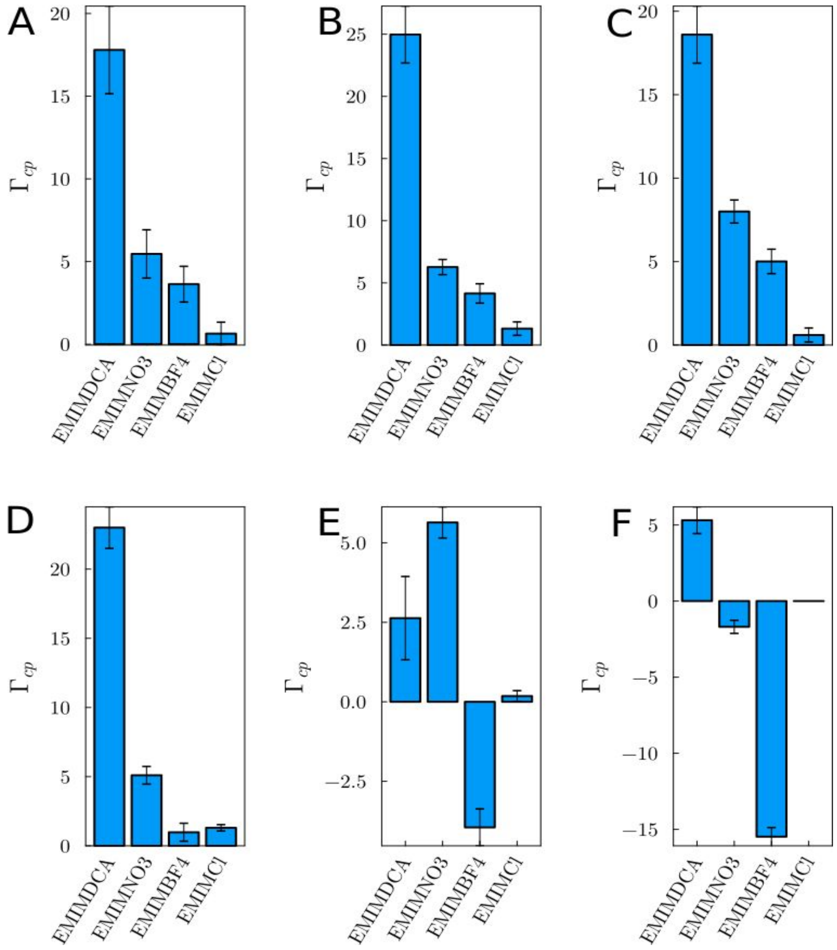

Figure S7. Visualization of IL preferential solvation parameters in single-IL systems at different concentrations: showcases solvation parameters for ionic liquids (ILs) in six distinct concentrations, with A) 0.5, B) 1.0, C) 1.5, D) 2.0, E) 2.5, and F) 3.0 mol L^{-1} solutions, providing a comparative analysis of IL behavior across varying solute concentrations.

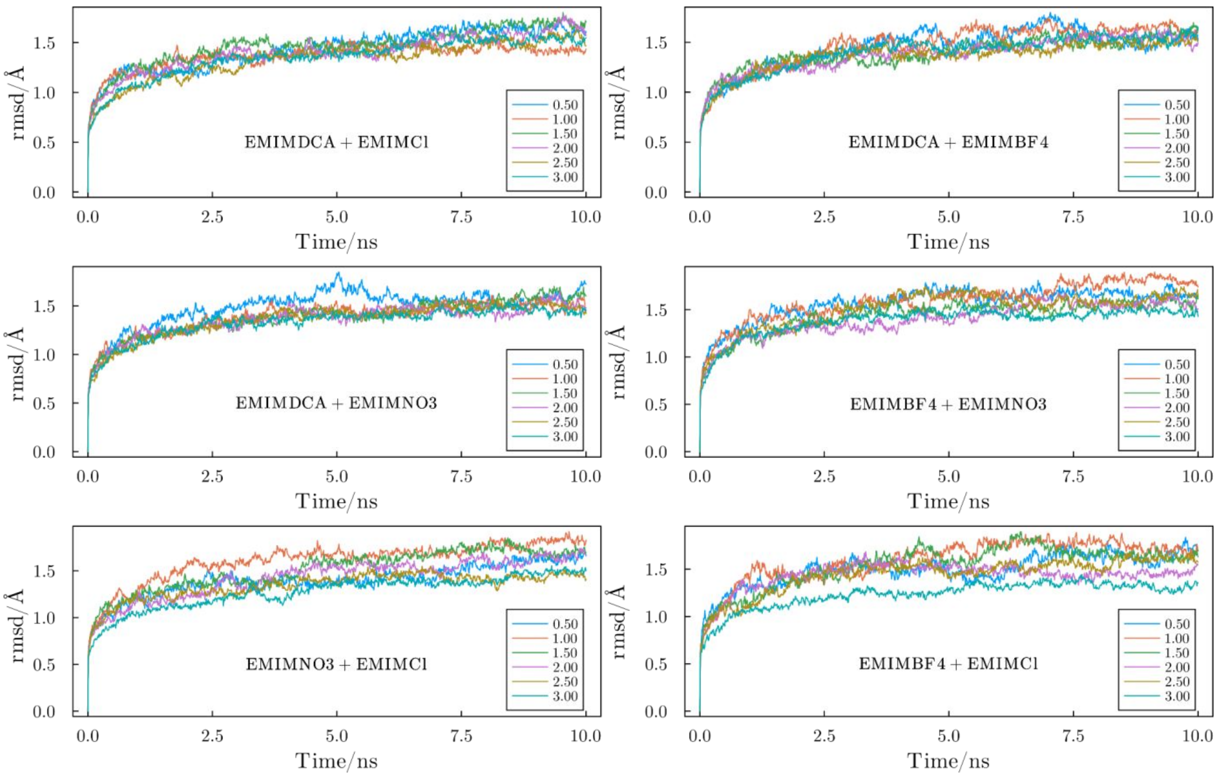

Figure S8. Protein root-mean-square deviation (RMSD) throughout the simulations. The average RMSD for 20 replicas is shown for each system. RMSD values consistently remain around 1.5 Ångströms for the protein backbone. This steady fluctuation within a confined range highlights the minimal structural changes the protein undergoes, ensuring its stability and retention within the native conformational ensemble.

Table S4. Evaluation of Lennard-Jones and Coulomb interactions between proteins and ions, for species positioned with at least one atom from a protein atom closer than 10Å (molecules are not broken-apart in this computation by a strict cutoff). Calculations were conducted using the SolventShellInteractions.jl package, developed by our group, which processes output files from GROMACS. Reported energies include standard deviations across all replicas. The table categorizes anions into two types: anion 1 and anion 2, representing the respective anions from the first and second ionic liquids in each studied mixture. For clarity, within the mixture "EMIMDCA + EMIMCl," DCA is denoted as Anion 1, and Cl as Anion 2, illustrating the labeling convention used.

Ionic Liquid mixture	RC (mol L^{-1})	Cation energy of interaction (kJ mol^{-1})		Anion 1 energy of interaction (kJ mol^{-1})		Anion 2 energy of interaction (kJ mol^{-1})	
		Q	LJ	Q	LJ	Q	LJ
EMIMDCA + EMIMCl	0.50	-140 ± 7	-303 ± 1	-1299 ± 10	-276 ± 1	-195 ± 4	1.0 ± 0.1
	1.00	-171 ± 13	-538 ± 1	-2135 ± 12	-383.1 ± 0.9	-217 ± 5	2.3 ± 0.2
	1.50	-240 ± 11	-690 ± 1	-2079 ± 10	-408.3 ± 0.9	-398 ± 7	7.9 ± 0.3
	2.00	-401 ± 13	-794 ± 1	-2279 ± 15	-448 ± 1	-669 ± 9	13.2 ± 0.3
	2.50	-672 ± 8	-821 ± 1	-1371 ± 11	-383 ± 1	-1236 ± 10	20.4 ± 0.5
	3.00	-622 ± 10	-989 ± 2	-2004 ± 13	-439 ± 1	-1086 ± 12	29.8 ± 0.5
	0.50	-520 ± 8	-188 ± 1	-343 ± 7	-36.1 ± 0.4	-295 ± 7	0.4 ± 0.2
	1.00	-521 ± 6	-356 ± 1	-770 ± 8	-79.5 ± 0.4	-482 ± 7	2.9 ± 0.2
	1.50	-755 ± 8	-540 ± 1	-796 ± 8	-99.9 ± 0.5	-677 ± 8	8.0 ± 0.3

EMIMBF4 + EMIMCl	2.00	-948 ± 9	-680 ± 1	-1033 ± 10	-123.3 ± 0.4	-1007 ± 10	12.8 ± 0.4
	2.50	-1152 ± 11	-817 ± 1	-838 ± 12	-135.5 ± 0.4	-1617 ± 12	28.6 ± 0.5
	3.00	-1256 ± 10	-1011 ± 1	-952 ± 10	-165.2 ± 0.4	-1597 ± 9	41.4 ± 0.6
EMIMNO3 + EMIMCl	0.50	-542 ± 6	-227 ± 1	-700 ± 5	-79.4 ± 0.5	-260 ± 5	3.7 ± 0.2
	1.00	-667 ± 8	-404 ± 1	-854 ± 7	-116.7 ± 0.8	-601 ± 8	5.6 ± 0.3
	1.50	-649 ± 8	-533 ± 1	-1255 ± 8	-149.2 ± 0.6	-671 ± 9	8.1 ± 0.3
	2.00	-585 ± 8	-702.1 ± 0.9	-1540 ± 11	-180.3 ± 0.7	-1095 ± 10	11.4 ± 0.4
	2.50	-782 ± 12	-871 ± 1	-1435 ± 13	-213.2 ± 0.8	-1502 ± 11	24.1 ± 0.4
	3.00	-968 ± 9	-1020.4 ± 0.9	-1396 ± 15	-203.3 ± 0.8	-1873 ± 12	38.4 ± 0.6
EMIMDCA + EMIMBF4	0.50	-155 ± 7	-284 ± 1	-980 ± 8	-219.0 ± 0.9	-193 ± 6	-18.2 ± 0.3
	1.00	-1± 10	-494 ± 1	-1759 ± 11	-348 ± 1	-272 ± 7	-33.3 ± 0.3
	1.50	17 ± 11	-625 ± 2	-1984 ± 12	-390 ± 1	-326 ± 7	-44.7 ± 0.3
	2.00	-137 ± 13	-752 ± 2	-2029 ± 12	-396 ± 1	-421 ± 9	-62.2 ± 0.4

	2.50	-262 ± 11	-812 ± 1	-1868 ± 14	-382 ± 2	-650 ± 12	-83.6 ± 0.5
	3.00	-460 ± 16	-861 ± 2	-1600 ± 16	-382 ± 1	-773 ± 9	-92.6 ± 0.4
EMIMDCA + EMIMNO3	0.50	-338 ± 9	-312 ± 1	-873 ± 10	-232 ± 1	-305 ± 6	-36.7 ± 0.5
	1.00	-214 ± 12	-554 ± 1	-1619 ± 12	-332 ± 1	-459 ± 8	-60.4 ± 0.6
	1.50	-518 ± 11	-625 ± 2	-2526 ± 11	-346 ± 1	-825 ± 9	-80.7 ± 0.6
	2.00	-369. ± 13	-757 ± 2	-1695 ± 15	-377 ± 1	-702 ± 10	-92.3 ± 0.6
	2.50	83 ± 15	-815 ± 2	-1862 ± 12	-341 ± 1	-1424 ± 15	-135.3 ± 0.8
	3.00	4 ± 10	-908 ± 2.	-1995 ± 13	-337 ± 2	-1215 ± 13	-135.1 ± 0.9
EMIMBF4 + EMIMNO3	0.50	-410 ± 7	-213 ± 1	-214 ± 5	-30.4 ± 0.3	-478 ± 8	-478 ± 8
	1.00	-392 ± 11	-392 ± 2	-625 ± 10	-58.1 ± 0.4	-1024 ± 10	-102.6 ± 0.6
	1.50	-324 ± 12	-499 ± 1	-585 ± 8	-69.0 ± 0.5	-1383 ± 12	-138.2 ± 0.6
	2.00	-817 ± 10	-676 ± 2	-636 ± 10	-89.1 ± 0.6	-1307 ± 12	-151.6 ± 0.9
	2.50	-618 ± 15	-724 ± 3	-962 ± 15	-103.6 ± 0.8	-1313 ± 13	-160 ± 1
	3.00	-1055 ± 12	-923 ± 2	-649 ± 11	-103.6 ± 0.5	-1583 ± 13	-205.4 ± 0.8

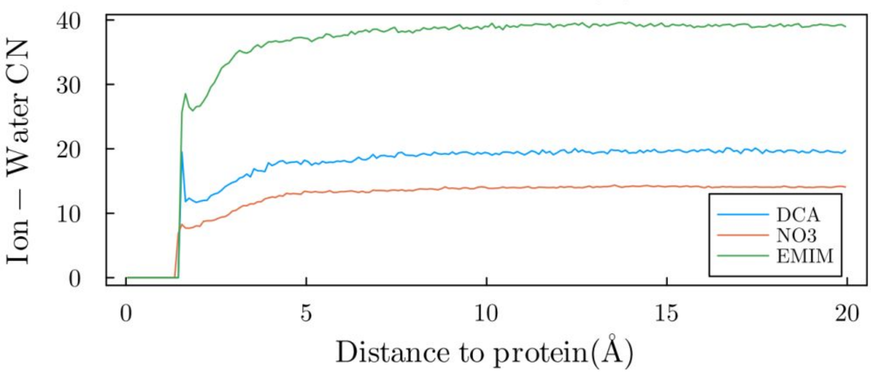

Figure S9. example of the computation of the water coordination numbers of DCA, NO3 and EMIM, as a function of the distance to the protein, in the system with DCA and NO3 at 2.0 mol L^{-1}. The CN reported consists of the number of water molecules within 5Å of the ions, per ion.

Table S5. DCA - water coordination numbers far from the protein. These are the number of water molecules within 5.0Å of the anion, considering the DCA molecules that have no atom within 20Å from the protein surface. RC is the reference IL concentration, and the anions present in each system are shown. The cation is EMIM in all systems. The data shown is the average and standard deviation of 20 replicas for each system.

RC \ system	DCA	DCA + Cl	DCA + NO3	DCA + BF4
0.5	30.1 ± 0.9	28.8 ± 0.9	30.4 ± 0.4	29.1 ± 0.9
1.0	23.5 ± 1.3	25.0 ± 0.4	26.0 ± 0.4	24.6 ± 0.7
1.5	21.7 ± 0.4	21.3 ± 0.4	22.8 ± 1.3	21.6 ± 0.3
2.0	18.9 ± 0.4	18.2 ± 1.3	20.0 ± 0.9	18.8 ± 0.8
2.5	17.2 ± 0.4	16.0 ± 1.8	17.0 ± 0.4	16.4 ± 0.7
3.0	14.6 ± 0.9	13.3 ± 0.9	15.2 ± 0.4	13.9 ± 0.6

Table S6. DCA - water coordination numbers far from the protein. These are the number of water molecules within 10.0Å of the anion, considering the DCA molecules that have no atom within 30Å from the protein surface. RC is the reference IL concentration, and the anions present in each system are shown. The cation is EMIM in all systems. The data shown is the average and standard deviation of 20 replicas for each system.

RC \ system	DCA	DCA + Cl	DCA + NO3	DCA + BF4
0.5	185.5 ± 0.5	186.9 ± 0.4	187.3 ± 0.5	185.5 ± 0.5
1.0	162.5 ± 0.4	167.0 ± 0.8	168.4 ± 0.6	165.2 ± 0.5
1.5	144.4 ± 0.4	148 ± 1	151.6 ± 0.4	147.0 ± 0.4
2.0	128.3 ± 0.3	132 ± 1	135.4 ± 0.4	128.7 ± 0.4
2.5	116.7 ± 0.5	117.5 ± 0.8	118.4 ± 0.4	112.7 ± 0.4

RC \ system	DCA	DCA + Cl	DCA + NO3	DCA + BF4
0.5	185.5 ± 0.5	186.9 ± 0.4	187.3 ± 0.5	185.5 ± 0.5
1.0	162.5 ± 0.4	167.0 ± 0.8	168.4 ± 0.6	165.2 ± 0.5
3.0	98.5 ± 0.3	102.3 ± 0.5	103.1 ± 0.3	95.6 ± 0.2

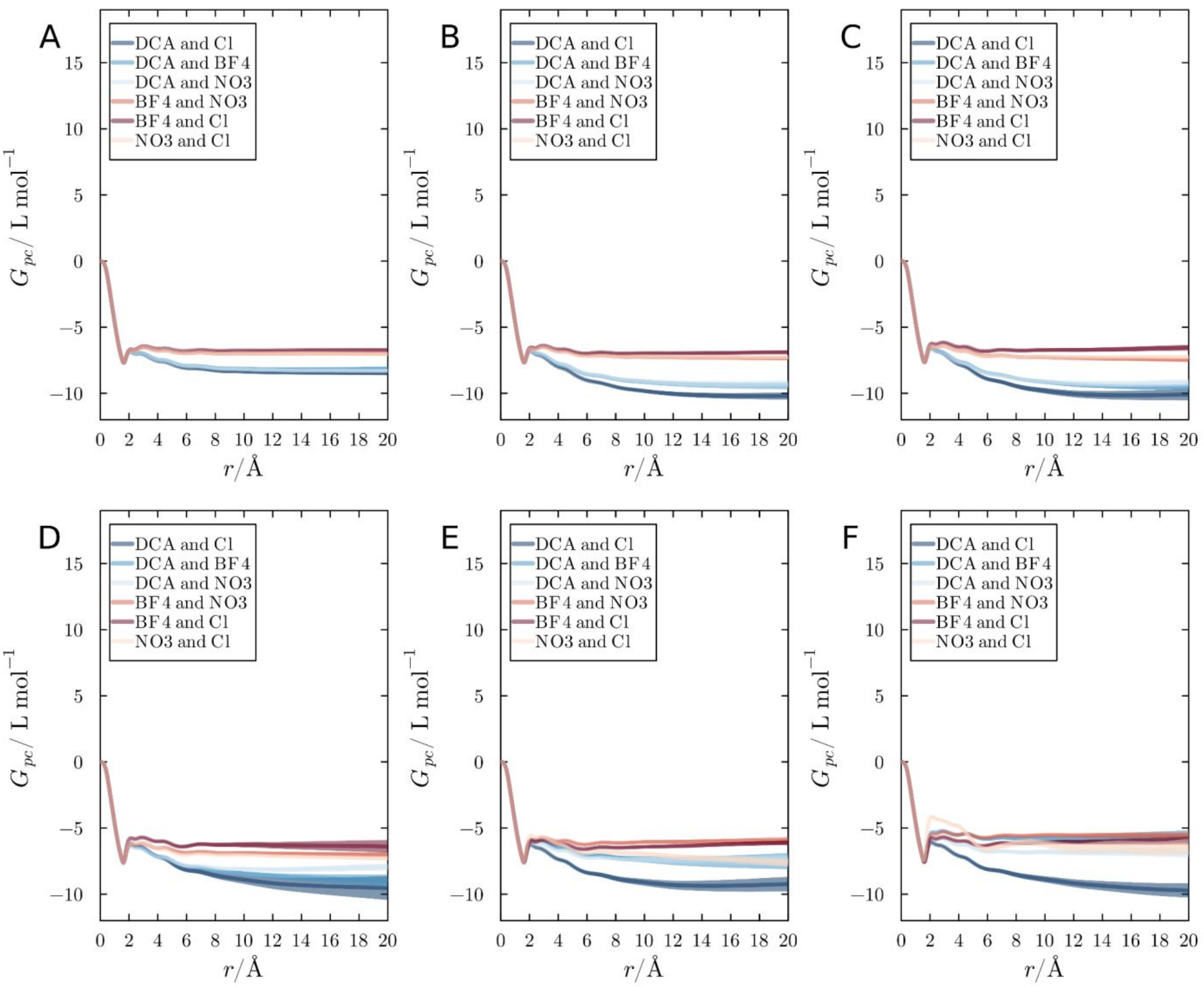

Figure S10. Water Kirkwood-Buff integrals for all simulated systems with two types of anions. The integrals are calculated over lengths that ensure proper convergence for the majority of the systems. Subfigures contain the total KB integral for the anions in systems with A) 0.50, B) 1.00, C) 1.50, D) 2.00, E) 2.50 and F) 3.00 mol L^{-1} IL concentration.
